# Sodium-coupled glucose transport, the SLC5 family, and therapeutically relevant inhibitors: from molecular discovery to clinical application

**DOI:** 10.1007/s00424-020-02433-x

**Published:** 2020-08-07

**Authors:** Gergely Gyimesi, Jonai Pujol-Giménez, Yoshikatsu Kanai, Matthias A. Hediger

**Affiliations:** 1 Membrane Transport Discovery Lab, Department of Nephrology and Hypertension, and Department of Biomedical Research, Inselspital, University of Bern, Kinderklinik, Office D845, Freiburgstrasse 15, CH-3010 Bern, Switzerland; 2 Department of Bio-system Pharmacology, Graduate School of Medicine, Osaka University, Osaka, Japan

**Keywords:** Glucose transport, SGLT1, SGLT2, SLC5 family, Diabetes, SGLT2 inhibitors, Gliflozins, Molecular docking, Tubuloglomerular feedback, Renin-angiotensin system, Nephroprotective, Cancer, Drug delivery

## Abstract

Sodium glucose transporters (SGLTs) belong to the mammalian solute carrier family SLC5. This family includes 12 different members in human that mediate the transport of sugars, vitamins, amino acids, or smaller organic ions such as choline. The SLC5 family belongs to the sodium symporter family (SSS), which encompasses transporters from all kingdoms of life. It furthermore shares similarity to the structural fold of the APC (amino acid-polyamine-organocation) transporter family. Three decades after the first molecular identification of the intestinal Na^+^-glucose cotransporter SGLT1 by expression cloning, many new discoveries have evolved, from mechanistic analysis to molecular genetics, structural biology, drug discovery, and clinical applications. All of these advances have greatly influenced physiology and medicine. While SGLT1 is essential for fast absorption of glucose and galactose in the intestine, the expression of SGLT2 is largely confined to the early part of the kidney proximal tubules, where it reabsorbs the bulk part of filtered glucose. SGLT2 has been successfully exploited by the pharmaceutical industry to develop effective new drugs for the treatment of diabetic patients. These SGLT2 inhibitors, termed gliflozins, also exhibit favorable nephroprotective effects and likely also cardioprotective effects. In addition, given the recent finding that SGLT2 is also expressed in tumors of pancreas and prostate and in glioblastoma, this opens the door to potential new therapeutic strategies for cancer treatment by specifically targeting SGLT2. Likewise, further discoveries related to the functional association of other SGLTs of the SLC5 family to human pathologies will open the door to potential new therapeutic strategies. We furthermore hope that the herein summarized information about the physiological roles of SGLTs and the therapeutic benefits of the gliflozins will be useful for our readers to better understand the molecular basis of the beneficial effects of these inhibitors, also in the context of the tubuloglomerular feedback (TGF), and the renin-angiotensin system (RAS). The detailed mechanisms underlying the clinical benefits of SGLT2 inhibition by gliflozins still warrant further investigation that may serve as a basis for future drug development.

## Expression cloning to unveil the molecular structure and function of sodium glucose cotransporters

The concept that transport of glucose across the intestinal brush border membrane (BBM) requires an active mechanism that is achieved through coupling of transport to the inwardly directed Na^+^ gradient has already been disclosed in 1960 [26]. It was subsequently refined and extended to cover active transport of a variety of molecules, including nutrients, neurotransmitters, metabolites, and electrolytes. These transporters are called cotransporters or symporters and they usually link uphill solute transport to the cotransport of Na^+^ or H^+^ [70]. The primary sequences of the corresponding transporters, however, remained unknown until later in 1980, when expression cloning approach was discovered, because the hydrophobic nature of these integral membrane proteins precluded their isolation in a form suitable for amino acid sequencing. The needed approach was conceptualized based on the observations that micro-injection of mRNA from rabbit small intestine into *Xenopus laevis* oocytes (X. oocytes) stimulated Na^+^-dependent and phlorizin-sensitive uptake of ^14^C-α-methyl-d-glucopyranoside (^14^C-α-MG), the specific substrate of the intestinal sodium-glucose cotransporter and that micro-injection of size-fractionated intestinal poly(A) RNA of about 2.4 kb specifically induced this transport function [68]. The X. oocyte expression system offered a convenient method for this purpose, as the relatively large cells (~ 0.8–1.3 mm diameter) easily allowed the micro-injection of poly(A) RNA (or cRNA derived from cDNA clones), and 3 days after injection, the encoded transporters could already be detected in the oocyte cell membrane using radio-isotope uptake studies or two-electrode voltage clamping (TEVC). A cDNA transcription library was then generated from the active size fraction, cDNA clones were screened functionally for their ability to induce uptake of ^14^C-α-MG, and a positive clone was identified, sequenced, and fully characterized in terms of its structure, function, and physiological roles [67]. Soon thereafter, the approach resulted in the identification of the primary structure of the human intestinal Na^+^/glucose cotransporter SGLT1 (SLC5A1) [67]. During the following years, expression cloning became the premier method to isolate transporter clones, as it did not require any DNA or antibody probes to screen cDNA libraries, only transporter-specific functional assays [141]. Indeed, this approach has revealed the structural and mechanistic foundations for transporters of iron (SLC11A2/DMT1/DCT1) [61], vitamin C (SLC23A1/SVCT1 and SLC23A2/SVCT2) [124], urea (SLC14A2/UT1) [206], glutamate (SLC1A1/EAAC1) [75], dibasic amino acids (SLC3A1/D2) [197], oligopeptides (SLC15A1/PepT1) [42], myoinositol (SLC5A3/SMIT) [90], iodide (SLC5A5/NIS) [27], and the epithelial calcium channel TRPV6/CaT1 [124]. The human homolog of rabbit SGLT1 (SLC5A1) [69] and the kidney-specific human homolog SGLT2 (SLC5A2) [76, 198] were subsequently identified. Using expression cloning with co-expression of SGLT2, an important activator, named MAP17, of SGLT2 was furthermore identified by expression cloning [23]. Overall, it soon became apparent that the sodium glucose cotransporters (SGLTs) belonged to a larger family of transporters, including members in lower organisms such as the *Escherichia coli* Na^+^/proline cotransporter PutP. Three decades after the initial cloning of the rabbit intestinal Na^+^-glucose cotransporter SGLT1, many new discoveries have been made in the field, from mechanistic analysis to molecular genetics, structural biology, drug discovery, and clinical applications. All of these advances have greatly shaped physiology and medicine [202].

## Brief description of key properties, expression patterns, physiological and pathological implications of SLC5 family members

**Fig. 1:**
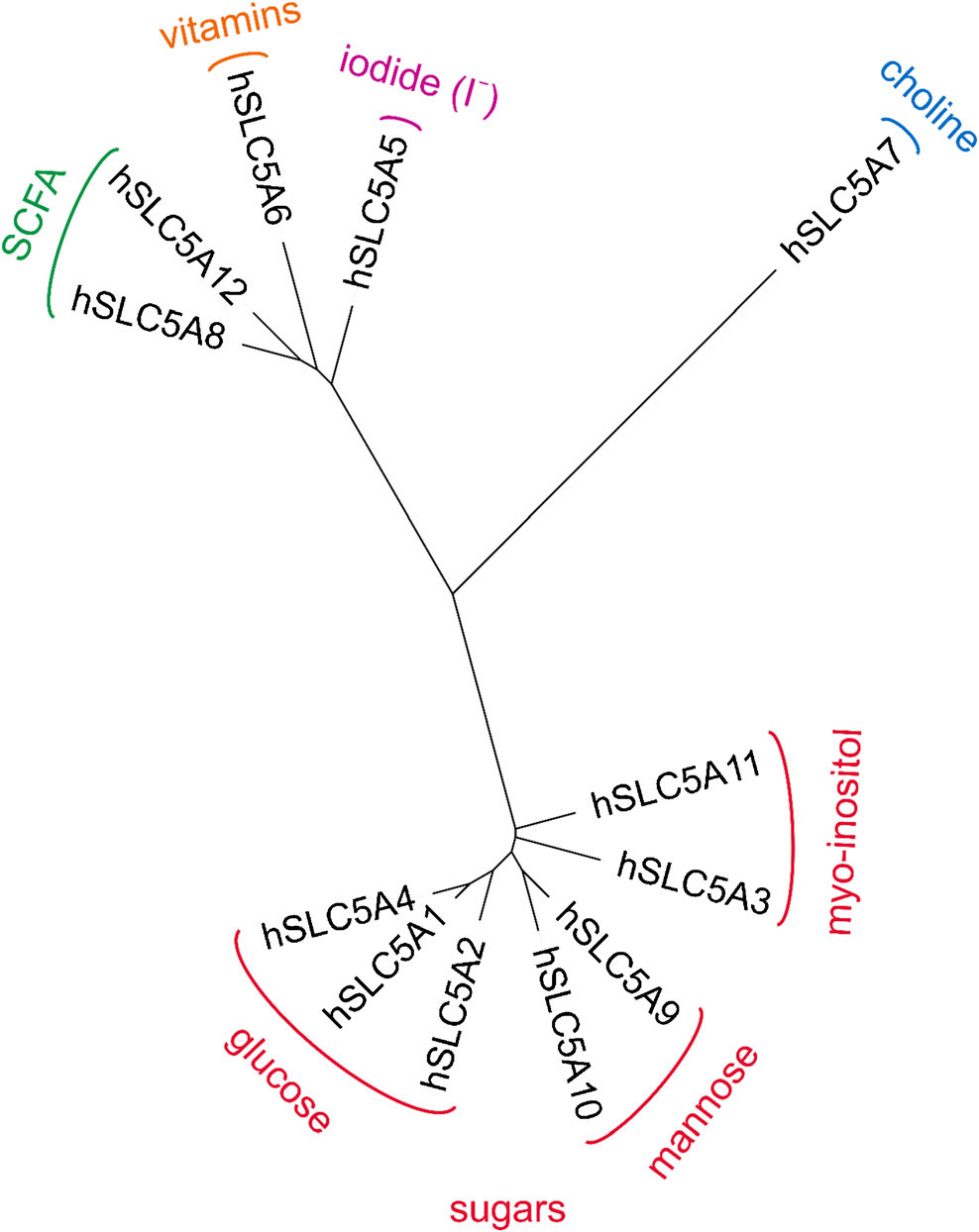
Phylogenetic tree of human SLC5 members. The physiological substrates of individual transporters are indicated (SCFA: short chain fatty acids). The phylogenetic tree was visualized with the Interactive Tree of Life (iTOL) server [95]

The human SLC5 solute carrier family includes 12 members. It is part of the sodium symporter family (SSS) that encompasses members from all kingdoms of life. The SSS family is also annotated in TCDB [147] as family #2.A.21, and the corresponding transmembrane domain in Pfam database [37] as “SSF” (“sodium:solute symporter family,” Pfam ID: PF00474). SLC5 members typically transport small solutes, such as sugars, vitamins, amino acids, or smaller organic ions such as choline or monocarboxylates (short chain fatty acids). Proposed evolutionary relationships between human SLC5 members are shown in Fig. 1. Sequences of the 12 human SLC5 members were downloaded from the UniProt database [181], and aligned by Clustal Omega [159, 160]. Based on this multiple alignment, a phylogenetic tree was generated using PhyML 3.0 with smart model selection (SMS) [60, 94] with default settings (Fig. 1). The resulting tree shows a partitioning of human SLC5 proteins in accordance with substrate selectivity, whereby the sugar transporters appear to be more closely related to each other than to non-sugar transporters. Human SLC5A7, which is a Na^+^/Cl^−^-coupled choline transporter, seems to be more distantly related to other SLC5 members.

### SLC5A1/SGLT1

SGLT1 was the first member of the SLC5 family to be cloned [67, 69] and it has been extensively studied during the past 4 decades [66, 71, 200, 202]. Its main function is the absorption of glucose and galactose across the intestinal brush-border membrane. However, as outlined later on in this review, it also plays a role in the reabsorption of these sugars in the kidney, where it can be found in the regions S2 and S3 of the proximal tubules [64]. Mutations in this gene lead to the intestinal glucose/galactose malabsorption (GGM, OMIM #606824), a rare metabolic disorder that causes a severe diarrhea that can be fatal unless glucose and galactose are removed from the diet [19, 201]. Moreover, as reflected by its renal function, GGM patients present mild renal glucosuria (see below), urinary tract infections, and calculus formation [19, 200]. Due to the pivotal role of SGLT1 in glucose absorption in the intestine, which is accompanied by water absorption, a treatment promoting the activity of SGLT1, named as oral rehydration therapy (ORT), is one of the standard medical procedures to prevent and overcome secretory diarrhea-related complications [71]. Recently, SGLT1 has been proposed to contribute to a new variety of physiological processes, including glucose sensing in the brain [202], protection against pathogens in activated lymphocytes [10], or embryonic implantation [151].

SGLT1 transports the natural sugars glucose and galactose, but not fructose or mannose. It is also able to transport some non-metabolized glucose analogues, such as α-MG and 3-O-methyl-glucose, but not 2-deoxy-glucose [71]. Regarding the transport mechanism, sugar transport requires the cotransport of Na^+^, which occurs with a stoichiometry of 2 Na^+^ ions per each transported sugar molecule [21, 92]. In addition, it has been proposed that SGLT1 can work as Na^+^ uniporter, and even as a water and urea channel [71].

### SLC5A2/SGLT2

SGLT2 is located in the apical membrane of the early renal proximal convoluted tubule S1 segments [207], where it mediates the absorption of most of the glucose present in the glomerular filtrate [76, 184, 198]. Mutations in the gene encoding SGLT2 are responsible for familial renal glucosuria (FRG, OMIM #233100) [109, 174], a disorder that results in loss of glucose in the urine despite normal blood glucose levels [19]. In fact, due to its highly specialized role in proximal tubule glucose reabsorption, SGLT2 has been largely studied by the pharmaceutical industry as a therapeutic target to control glucose levels in diabetic patients [155, 170], as will be explained later on in detail. Moreover, SGLT2-targeting drugs have received increasing attention due to their cardioprotective effects in diabetic patients and, thus, possible additional use as pharmaceutical tools to prevent heart failure [187]. While the expression of SGLT2 is mainly restricted to the early proximal tubules of the kidney, interestingly, its expression has been detected in pancreas, prostate tumors, and glioblastoma, which opens the door to potential new strategies for cancer treatment by targeting SGLT2 in those tissues [202].

Numerous in vivo experiments in the 1980s highlighted the existence of a low-affinity glucose transport system in the early proximal tubules [7]. Subsequently, SGLT2 was successfully cloned and characterized in the early 1990s [76, 198]. However, due to the low signal-to-noise ratio observed for protein activity in the different expression systems tested [71, 76, 207], an accurate and comprehensive description of its transport mechanism remained elusive for more than 2 decades. Nevertheless, recent studies revealed that co-expression of SGLT2 with MAP17, a small protein that interacts with SGLT2, greatly intensifies its transport function [23]. This finding allowed to confirm previous observations [71] that SGLT2 is a low-affinity high-capacity transporter, very selective for glucose, inhibited by phlorizin and that the Na^+^ to glucose coupling stoichiometry is 1:1 [23]. Therefore, mechanistic details of SGLT2 transport function is expected to follow soon, which will improve our molecular understanding of its physiological and pharmacological properties.

### SLC5A4/SGLT3 (also known as SAAT1)

Expression of human SGLT3 has been described in enteric neurons of the intestinal epithelia and in neuromuscular junctions of the skeletal muscle [35]. Interestingly, when expressed in X. oocytes, this protein has shown to be unable to transport glucose, while the binding still induces currents through SGLT3-mediated depolarization of the plasma membrane resting potential. SGLT3 binds glucose with low affinity (*K*_m_ = 20 mM), while galactose, fructose, and mannitol do not interact with the protein. The substrate-induced currents through SGLT3 are Na^+^-dependent, increase in the presence of H^+^, and are specifically inhibited by phlorizin [35]. Imino sugars were described as potent and specific activators of the electrogenic properties of SGLT3. Interestingly, imino sugars are also used to treat type 2 diabetes and lysosomal storage diseases based on their inhibition of α-glucosidases and glucosyltransferases [211]. Imino sugars may serve as tool compounds to explore the precise physiological role of SGLT3 [190]. Due to its location in the enteric neurons and its particular transport mechanism, it has been proposed that SGLT3 works as a glucose sensor, the activity of which might regulate the intestinal motility in response to glucose [35]. Likewise, it was speculated that it could regulate skeletal muscle activity by depolarizing neuromuscular junction cells in response to glucose [35]. Recent studies provided evidence for expression of SGLT3 in human kidney, where it might contribute to Na^+^ transport in proximal tubules [88]. Also, whole-exome sequencing identified a genetic variant of SGLT3, which disrupts glucose-induced sodium conductance, and is present in some patients affected by attention deficit/hyperactivity disorder (ADHD). The co-segregation of the SGLT3 variant and ADHD phenotype was, however, imperfect [154]. Taken together, while the functional properties of SGLT3 have been studied by several investigators, its physiological role is still the subject of debate [165]. The glucose-sensing mechanism that has been proposed based on in vitro studies, showing that SGLT3 generates membrane currents in the presence of high concentrations of glucose, is lacking in vivo validation. Also, the tissue localization of this protein needs to be clarified to help predict its physiological role. In addition, the role of imino sugars should be further investigated. The lack of a human disease phenotype linked to the transporter malfunction and the absence of appropriate transgenic animal models and inhibitors further slowed down defining its true physiological role.

### SLC5A9/SGLT4

The expression of SGLT4 in humans has been detected in the small intestine and kidney [171]. Functional studies using COS-7 cells overexpressing SGLT4 revealed that it is a Na^+^-dependent α-MG transporter (*K*_m_ = 2.6 mM). Moreover, transport of α-MG was inhibited by mannose and glucose, and to a lower extent by fructose and the metabolite 1,5-anhydro-d-glucitol (1,5-AG). Direct measurements of mannose uptake by SGLT4 and the capacity of mannose to inhibit SGLT4-mediated α-MG transport (IC_50_ = 0.15 mM) indicated that SGLT4 is a Na^+^-coupled mannose transporter [171]. Three rare variants of this gene were identified in patients of proliferative diabetic retinopathy and it was suggested that this protein is expressed in retinal endothelial cells, where it may play a role in the pathogenesis of this disease [180]. Recent genetic studies revealed that SGLT4 is expressed in kidney-pancreatic-colorectal tumors, while not expressed in the matched normal tissues [49]. Similarly, a genome-wide association study identified a SNP near the SGLT4 locus that affects susceptibility for colorectal cancer development [44]. Overall, the lack of detailed functional information, together with limited information regarding its expression profile, results in a poorly understood physiological role of this transporter.

### SLC5A10/SGLT5

SGLT5 has been shown to be highly expressed in human kidney [58]. Immunolocalization revealed apical expression in the proximal straight tubules [54]. Functional studies using SGLT5-overexpressing TREX HEK293 cells revealed that it is a mannose (*K*_m_ = 0.45 mM) and fructose (*K*_m_ = 0.62 mM) transporter, which shows the typical functional characteristics of the SLC5 glucose transporter family members, such as the inhibition by phlorizin and Na^+^-dependence of the transport process [58]. Due to its selectivity toward fructose and mannose, it has been speculated that it is responsible for reabsorption of these sugars from the glomerular filtrate. In support of this idea, studies of Slc5a10 knockout mice showed an increased loss of fructose in the urine, without affecting the plasma levels of fructose [46]. Furthermore, studies of the renal reabsorption of fructose in rat proximal tubules revealed that this process can be blocked to a large extent by phlorizin and that it is Na^+^-dependent [54]. In addition, recent genome-wide association studies revealed that genetic variations of SGLT5 are associated with impaired 1,5-anhydroglucitol (1,5-AG) blood levels [96]. 1,5-AG is a monosaccharide found in nearly all foods and its blood concentration decreases during times of hyperglycemia and, within a couple of weeks, returns to normal levels in the absence of hyperglycemia. Rare loss-of-function mutations of SGLT5 have been associated with lower levels of 1,5-AG, indicating that SGLT5 reabsorbs this monosaccharide [100]. In healthy individuals, 1,5-AG level is kept relatively constant through intestinal absorption and renal reabsorption. Interestingly, 1,5-AG reabsorbed by the kidney is inhibited by competition with glucose [203]. Thus, during hyperglycemia, when the kidney cannot reabsorb all glucose via SGLT1 and SGLT2, this leads to inhibition of 1,5-AG reabsorption, presumably via SGLT5, and therefore a decrease in the of 1,5-AG levels in the blood. Once the hyperglycemia is corrected, 1,5-AG begins to be reabsorbed from the kidney back into the blood at a steady rate and if a person’s glucose levels remain below 10 mM for approximately 4 weeks, 1,5-AG will return to its normal levels. The 1,5-AG test is currently FDA-approved for diabetes patients to measure 1,5-AG levels in the blood, in order to determine the history of hyperglycemic episodes [203], complementary to HbA1c and fructosamine tests. Despite all these interesting recent findings, only limited information is available about the role of the SGLT5 transporter in health and disease and to what extent its functional activity affects the 1,5-AG diabetes test. SGLT2 inhibitors, which lower blood glucose and produce glycosuria, may likely cause abnormal circulating 1,5-AG levels and, thus, could interfere with the diabetes test.

### Other SLC5 family members

The SLC5 family comprises 12 members, including the abovedescribed sugar transporters (SGLT1-5), in addition to transporter for other substrates, such as the myo-inositol (SLC5A3 and SLC5A11), iodide (SLC5A5), monocarboxylate (SLC5A8 and SLC5A12), choline (SLC5A7), and vitamin (SLC5A6) transporters. All these membrane proteins share a common transport mechanism, which uses the Na^+^ electrochemical gradient to sense (SGLT3) or incorporate their substrates into cells.

### SLC5A3/SMIT1 and SLC5A11/SMIT2

The SLC5A3 Na^+^/myo-inositol transporter, known as SMIT1, was found to be widely expressed in humans [9], with prominent expression in the intestine and brain [5]. In addition to myo-inositol (*K*_0.5_ = 50 μM), SMIT1 also transports d-glucose with low affinity [200]. Studies with SMIT1 knockout mice revealed that it plays an essential role in osteogenesis, bone formation, and maintenance of bone mineral density [28]. SMIT2 was identified as a major gene responsible for the syndrome of infantile convulsions and paroxysmal dyskinesia (ICCA syndrome), as well as for benign familial infantile convulsions (BFIC) [140]. As observed for SMIT1, SMIT2 is expressed in a wide variety of human tissues [140], but in contrast to SMIT1, SMIT2 does not transport glucose [97]. Genetic studies have shown that SMIT2 interacts with immune-related genes and it seems to be involved in certain immune effects. Accordingly, it was proposed that SMIT2 could function as an autoimmune modifier [176]. Recent studies revealed a strong correlation between the expression of the myo-inositol transporters SMIT1 and SMIT2 and psychiatric diseases, e.g. schizophrenia and bipolar disorder, and it was suggested that alterations in their expression in specific brain regions account for the symptoms of these diseases [188]. Another recent study showed an altered expression pattern of SMIT1 and SMIT2 in the sciatic nerve and dorsal root ganglia in an experimental diabetes model, which may play a role in the pathogenesis of diabetic neuropathy [41].

### SLC5A5/NIS

The Na^+^/iodide transporter SLC5A5, also known as NIS, is highly expressed in the thyroid gland, where it mediates accumulation of iodide (I^−^), which is required for the biosynthesis of the thyroid hormones T3 and T4 [30]. NIS is also expressed in non-thyroid tissues, such as salivary glands, stomach, lactating breast, and primary and metastatic breast cancer [136]. In addition, NIS is expressed in the small intestine, where it contributes to the absorption of dietary I^−^ [114]. In addition to I^−^, NIS also transports thiocyanate (SCN^−^) and chlorate (ClO_3_^−^) with a stoichiometry of 2 Na^+^:1 substrate. Interestingly, it also transports the pollutant perchlorate (ClO_4_^−^), although with a stoichiometry of 1:1 [123]. Mutations in the SLC5A5 gene lead to a condition known as I^−^ transport defect (ITD), which reduces the accumulation of I^−^ in the thyroid and results in hypothyroidism [136]. Due to its pivotal role in I^−^ absorption, NIS is the major target for diagnosis and therapy of thyroid cancer using iodide radioisotopes, whereby its expression level is crucial for tumor prognosis. The relatively high expression of NIS in breast cancer also makes it a potential target for the treatment of the disease [214].

### SLC5A8/SMCT1 and SLC5A12/SMCT2

SMCT1 is a high-affinity membrane transporter of lactate, and can also mediate the uptake of other monocarboxylates such as pyruvate, butyrate, propionate, and acetate [105]. SMCT1 expression can be found in the intestinal colon and the kidneys, and to a lower extent, in the brain and retina [48]. In the colon, SMCT1 is postulated to be responsible for the apical uptake of short-chain fatty acids (SCFA) such as acetate, propionate, and butyrate generated by bacterial fermentation of dietary fiber [11, 162]. SMCT2 is a low-affinity membrane transporter of lactate, also transporting other monocarboxylates, including pyruvate and nicotinate [55]. SMCT1 and SMCT2 are both Na^+^-coupled. SMCT1-mediated transport is electrogenic with a Na^+^ to SCFA stoichiometry of 2:1, whereas SMCT2-mediated transport is electroneutral (Na^+^ to SCFA stoichiometry of 1:1) [162]. Moreover, SMCT1 has been shown to function as a tumor suppressor gene for colon, thyroid, stomach, kidney, and brain tumors [48]. SMCT1 and SMCT2 are both expressed in the apical membranes of the intestine and kidneys. However, while SMCT1 is found mainly in colon and outer kidney cortex, SMCT2 is present in the proximal parts of the intestinal tract and both kidney cortex and medulla [166]. Kidney-specific ablation of the expression of SMCT1 and SMCT2 resulted in a marked increase in urinary loss of lactate and a decrease in blood levels, indicating that these transporters might be responsible for renal lactate reabsorption [172]. A recent study of protein-protein interactions revealed that the PDZK1 adaptor protein is a binding partner of both SMCT1 and SMCT2 and additionally identified a molecular complex of SMCT1-PDZK1 and the urate transporter URAT1 (SLC22A12) (see “transporter complex” in Fig. 4a on the right). This suggests a possible role of SMCT1 in urate reabsorption in the kidney [167]. Another quite recent work showed the expression of both SMCT1 and SMCT2 in pancreas and also that SMCT1 function can be regulated by insulin [101].

### SLC5A7/CHT1

CHT1 is a high-affinity Na^+^/choline transporter (*K*_m_ = 2 μM), which is exclusively expressed in tissues containing cholinergic neurons. There, its transport activity constitutes the rate-limiting step for acetylcholine synthesis [84, 118]. In contrast to the other members of the SLC5 family, the transport process mediated by CHT1 is Cl^−^-dependent and regulated by extracellular pH [72]. CHT1 resides in intracellular compartments and is translocated to the plasma membrane in response to neuronal activity [62]. CHT1 knockout mice have a normal embryonic development, but die after birth due to defective cholinergic neurotransmission [43]. Patients with truncating SLC5A7 mutations underlie a spectrum of dominant hereditary motor neuropathies [152]. A single nucleotide polymorphism in the SLC5A7 gene with high prevalence in the Asian population, resulting in a replacement of isoleucine by valine in the third transmembrane domain, has been reported to decrease the ability of CHT1 to transport choline by about 50%, thereby representing a risk factor for cholinergic dysfunction in this population [62].

### SLC5A6/SMVT

SMVT is a Na^+^-dependent transporter of the water-soluble vitamins pantothenic acid, biotin, and α-lipoic acid, the latter being a cofactor of several enzymes such as pyruvate dehydrogenase. Transport through SMVT is electrogenic and occurs with a stoichiometry of 2 Na^+^ to 1 vitamin molecule [177]. Like NIS (SLC5A5), to which it shows a high degree of sequence identity, SMVT transports I^−^ as well [29]. SMVT is ubiquitously distributed in the human body. However, stronger expression levels are found in absorptive tissues such as the intestine, the kidney, and the placenta [132]. Studies of intestine-specific Slc5a6 knockout mice indicated that SMVT is indispensable for intestinal biotin uptake [52]. In addition, SMVT was found to be required to keep a normal mucosal integrity [145]. Furthermore, it was shown that the defects due to the absence of SMVT in knockout mouse intestine can be reverted by biotin and pantothenic acid supplementation [146]. A recent study described a neurodegenerative disorder as consequence of a biallelic mutation in the SLC5A6 gene, which could be clinically improved by “triple vitamin” (biotin, pantothenate, and lipoate) replacement therapy [17]. Due to its broad tissue distribution and substrate range, SMVT is exploited as a drug delivery transport system to increase the bioavailability of prodrugs as conjugates of biological substrates of SMVT with drugs. For example, a biotinylated lipid prodrug of acyclovir has been generated for improved cellular uptake via SMVT in corneal epithelial cells, to be used for the treatment of herpes simplex virus keratitis [182].

## Description of the transport properties of SGLT1 to SGLT5

**Fig. 2:**
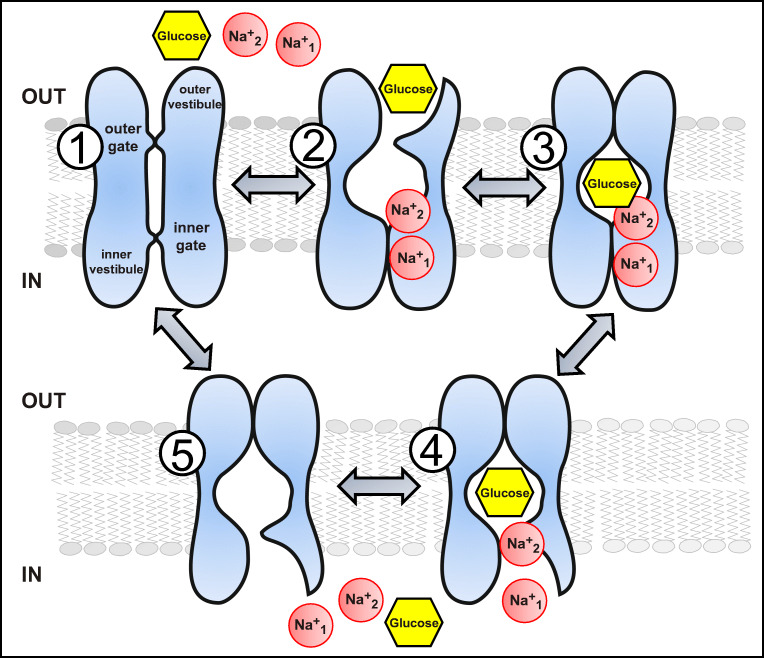
Na^+^-glucose cotransporter SGLT1 kinetic model. Extracellular Na^+^ binds first to the Na^+^_1_ and Na^+^_2_ binding sites of the empty carrier (states #1 and #2). This opens the external gate, allowing glucose to bind to the central pocket, whereupon the outer barrier closes to form the occluded state (#3). The internal barrier opens and the two Na^+^ ions and glucose can exit to the cytoplasm via the aqueous inner vestibule (#4). The transport cycle is then completed (#5) and the empty carrier returns to its original state (#1), ready for the next transport cycle. The transport rate of SGLT1 depends on the rate of the conformational changes needed to open and close the outer and inner barriers (step #2 to #3 and step #3 to #4, respectively)

The sodium-dependent glucose transporters or SGLTs are secondary active transporters present in the plasma membranes of different intestinal and renal epithelial cells. As already alluded to, they are able to transport their substrates against their concentration gradients, by using the energy provided by the inwardly directed Na^+^ electrochemical gradient generated by the Na^+^/K^+^-ATPase. Their transport process follows the so-called alternating access model [73], which means that the substrate binding site is alternately exposed to both sides of the plasma membrane (Fig. 2). Given the positive charge of the Na^+^ ions, the transport process is electrogenic, and therefore, induces depolarization of the plasma membrane resting potential. Thus, electrophysiological methods have been widely used to characterize the functional properties of these transporters [71]. These and other functional studies, together with the structural information obtained from the homolog vSGLT from *Vibrio parahaemolyticus* [40], have revealed the mechanistic details of the transport cycle of these transporters [31] and uncovered a kinetic model for the well-studied SGLT1 [202].

### SLC5A1/SGLT1

According to the latest version of this kinetic model for SGLT1 (Fig. 2) [202], there are five different steps within the transport cycle. In the first step, 2 extracellular Na^+^ ions bind the protein, which leads to the opening of the first of the two proposed structural gates, the external gate, allowing the transit of a glucose molecule through the external vestibule to a binding site located within the core of the protein (step 2). When both Na^+^ and glucose are bound, the external gate closes and the protein is in an occluded state, in which the binding site is not accessible from either sides of the membrane (step 3). In step 4, the inner gate opens, allowing the release of glucose and Na^+^ through the internal vestibule to the cytosol. Finally, to complete the transport cycle, the empty transporter returns to the initial conformation (step 5). This transport cycle has been shown to be reversible, and the direction and transport rate is dependent on the transmembrane concentration gradients of Na^+^ and the electric potential [133]. There is asymmetry in sugar kinetics and specificity between the forward and reverse transport modes, in line with the physiological role of the transporter to accumulate sugars within the cells [39]. As previously mentioned, the transport stoichiometry is 2 Na^+^:1 glucose per cycle for SGLT1, which has been determined by measuring the reversal potential of the transport-induced currents [21] and combining electrophysiological and radiotracer flux measurements [76, 102]. It is remarkable that in the absence of glucose, there is a specific Na^+^-leak current through SGLT1, indicating that this protein can also function as a Na^+^ uniporter. Moreover, it has been shown that SGLT1 can transport water passively. However, while the Na^+^ uniport requires conformational changes and is saturable, water permeation shows a behavior similar to water channels [98]. Interestingly, water permeation through SGLT1 is independent of Na^+^ and glucose concentration gradients, but can be blocked by phlorizin. This property has been recently validated experimentally, since it has been shown that water permeability through SGLT1 can be altered by mutating residues lining the sugar transport pathway [213]. Likewise, it has been shown that SGLT1 can transport urea, which is achieved through the same pathway as the water molecules [213]. In terms of the physiological roles of these permeabilities, as stated by the authors [213], the water permeability of SGLT1 is orders of magnitude lower than that of aquaporins, which may be partially corrected by the high density of the transporters in the intestinal brush border membrane. Likewise, the precise physiological role of SGLT1-mediated urea transport requires further examination. Another feature of the SGLT1-mediated transport mechanism that has been studied in detail is its pre-steady state kinetics. The transient movements of the transporter generate electric currents, which reflect the Na^+^ binding/release and/or the movement of charged/polar residues within the electrical field across the membrane in response to changes in the resting membrane potential of the cell [65, 66]. Electrophysiological studies revealed that the SGLT1 pre-steady state currents are blocked by external phlorizin and glucose, and that they are Na^+^-dependent, which indicates that Na^+^ binds the transporter in the absence of the sugar [65, 99]. As a consequence of the Na^+^-dependence of the pre-steady state movements, the distribution of the conformations of the SGLT1 population in the membrane reflects the availability of external Na^+^ [71].

### SLC5A2/SLGT2

As already noted, the relatively low functional activity of SGLT2 expressed in oocytes or cultured cells somewhat limited the study of the functional properties of this protein. Nevertheless, the studies clarified that SGLT2 corresponds to the previously described and long-awaited low-affinity high-capacity transporter of the kidney proximal tubule S1 segments [76]. Using expression studies in X. oocytes, it was demonstrated that human SGLT2 mediates saturable Na^+^-dependent and phlorizin-sensitive transport of D-glucose and α-MD with *K*_m_ values of 1.6 mM for α-MD and ~ 250 to 300 mM for Na^+^, consistent with the previously reported low affinity Na^+^/glucose cotransport. In contrast to SGLT1, SGLT2 did not transport D-galactose. By comparing the initial rate of ^14^C-α-MD uptake with the Na^+^ influx calculated from α-MD-evoked inward currents, it was shown that the Na^+^ to glucose coupling ratio of SGLT2 is 1:1. Using combined in situ hybridization and immunocytochemistry with tubule segment-specific marker antibodies, it was demonstrated that there is an extremely high level of SGLT2 message in proximal tubule S1 segments [76]. Subsequent studies, using HEK293T cells as an expression system, were in agreement with these findings. In addition, the affinity for d-glucose was determined and the *K*_m_ was 5 mM, confirming the low affinity of SGLT2 [71]. Phlorizin turned out to be a more potent inhibitor of SGLT2 (IC_50_ = 11 nM) than of SGLT1 (IC_50_ = 140 nM). As presented later in this review (see the “SGLT2 regulation” section), a recent study demonstrated that co-expression of SGLT2 together with the protein MAP17 greatly increases SGLT2 functional activity [23, 24].

### SLC5A4/SGLT3

When expressed in X. oocytes, SGLT3 did not show any ability to transport glucose despite being correctly inserted in the plasma membrane. As already mentioned, glucose was able to induce depolarization of the membrane potential of the SGLT3-expressing oocytes, which was reversible and inhibited by phlorizin. These electrophysiological properties indicated substrate selectivity among the different sugars tested, and only glucose and α-MG induced currents, albeit with very low affinity (*K*_0.5_ = 20 mM). The authors highlighted that no sugar-induced currents were observed in the absence of Na^+^, but currents were significantly increased by lowering the pH levels, suggesting permeation of H^+^ through SGLT3. Furthermore, due to the low activation energy when measuring the temperature dependence of the currents, the authors suggested that SGLT3 shares more similarity to ion channels than transporters [35]. Studies of the substrate selectivity of SGLT3 revealed that substrate binding happens with very low affinity, with *K*_m_ values ranging from 19 to 43 mM. In contrast, SGLT3 exhibited high-affinity for imino sugars, with *K*_m_ values ranging from 0.5 to 9 μM. Moreover, the later study revealed that phlorizin inhibits sugar-induced currents through SGLT3 (*K*_i_ = 0.12 mM) and hyperpolarized the membrane potential in the absence of sugar, suggesting a possible Na^+^-leak current through SGLT3 [190]. Strikingly, the mutation of a single amino acid converted SGLT3 into a sugar transporter with functional properties similar to those of SGLT1. Specifically, replacement of glutamate in position 457 by glutamine, the amino acid present in the homologous position for SGLT1 and SGLT2, turned SGLT3 into a transporter with broad selectivity for sugars, with much higher affinity for glucose, α-MG, and phlorizin, and a stoichiometry of 2 Na^+^:1 substrate. Furthermore, these studies pointed out the high conductance of H^+^ at acidic levels, and again posed the question of whether SGLT3 behaves like a cation uniporter or a channel-like transporter [12]. In order to understand the function of SGLT3 better, several studies have been conducted with rodent SGLT3 isoforms. Some of these proteins also acted as “glucose sensors.” However, significant differences were observed regarding response to sugars, phlorizin, and H^+^ [165]. Moreover, initial studies with the porcine SGLT3 indicated that this isoform acts as Na^+^-coupled glucose transporter [34]. Overall, as already noted in the previous section, there is no consistency for the functional properties of SGLT3 among species, and further studies are needed to reveal the in vivo functional properties.

### SLC5A9/SGLT4

Regarding SGLT4, there is only a single study describing the functional properties of this protein, and while it provides information about the substrate selectivity, as already described above, the description of the putative transport mechanism is limited to the mentioning of the Na^+^-dependence of the transport process [171].

### SLC5A10/SGLT5

Similarly, initial studies with SGLT5 defined its substrate selectivity and showed that the transport process was Na^+^-dependent [58]. In addition to that, a more recent study revealed that SGLT5-mediated sugar transport is electrogenic, sensitive to voltage, and the authors proposed a Na^+^:glucose coupling ratio of 1:1. Moreover, it was shown that in the absence of Na^+^, H^+^ could also drive glucose transport, even though to a lower extent. It is also interesting to mention that no pre-steady state currents were observed for SGLT5 [51]. As already noted, this transporter is of particularly interest in the context of the FDA-approved test to diagnose blood sugar levels in diabetes patients, which measures the plasma level of the metabolite 1,5-AG, since SGLT5 is likely responsible for the reabsorption of 1,5-AG in the proximal tubule of the kidney, and the transport is inhibited by glucose in response to hyperglycemia.

## SGLT1 and SGLT2 in transepithelial sugar transport in the intestine and kidney

**Fig. 3:**
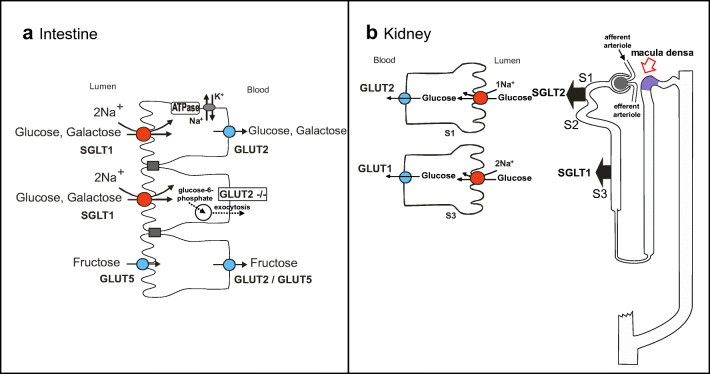
Na^+^/glucose cotransporters in the intestine (**a**) and kidney (**b**). SGLT1, Sodium Glucose Cotransporter (gene name: SLC5A1). SGLT2, Sodium Glucose Cotransporter (gene name: SLC5A2). **GLUT2,** “Facilitated” (passive) Glucose Transporter (gene name SLC2A1). **GLUT5**, “Facilitated” (passive) Glucose Transporter (gene name SLC2A5). SGLT1 is expressed in the brush border membrane of enterocytes and in the apical membranes of kidney of epithelial cells of the proximal straight tubules (second part of S2 segments and all of S3 segments). SGLT2 is expressed in the apical membranes of the kidney early proximal tubule cells (segment S1). GLUT2 is expressed in the basolateral membranes of intestine and renal proximal tubule S1 cells. In kidney proximal tubule S3 segments, cytosolic glucose exit occurs via basolateral GLUT1. In the intestine, in the absence of GLUT2, the current concept is that an alternative basolateral exit pathway exists, according to which glucose is converted to glucose-6-phosphate, which is transported into vesicles, followed by exocytosis [168]. GLUT5 is expressed in both apical and basolateral membranes of intestinal cells

In the small intestine (duodenum, jejunum), dietary carbohydrates are hydrolyzed to monosaccharides by pancreatic enzymes and brush-border hydrolases such as lactase and sucrase-isomaltase, resulting in high sugar concentrations on the brush border surface after a carbohydrate-rich meal. The digestion products are primarily d-glucose, d-galactose, and d-fructose, which must be efficiently absorbed by mature enterocytes in the upper one-third of intestinal villi, to avoid osmotic imbalance, as presented in Fig. 3a. In the kidney, d-glucose is freely filtered at the glomerulus and almost completely extracted from the tubular fluid by the proximal tubule transporters SGLT2 and SGLT1 (Fig. 3b), and returned to the blood. Approximately 90% of the filtered glucose is reabsorbed by the early S1 segment of the proximal tubules, and only a smaller fraction reaches the proximal straight tubule (later part of S2 segments and all of the S3 segments). As already noted, transport of each glucose molecule is coupled either to the cotransport of two Na^+^ ions (SGLT1) or one Na^+^ ion (SGLT2) (Fig. 3). Once inside the cell, glucose can diffuse into the blood via GLUT2 [109]. The Na^+^/K^+^-ATPase [163], located in the basolateral membrane, pumps Na^+^ out of the cell to maintain the inwardly directed Na^+^ electrochemical gradient required to drive uphill glucose transport across the brush border membrane. The high-affinity low-capacity Na^+^-glucose cotransporter SGLT1 is expressed in the small intestine, whereas expression of the low-affinity high-capacity Na^+^/glucose cotransporter SGLT2 is almost exclusively restricted to the early proximal tubule S1 segment of the kidney (besides some expression in pancreas and cancer tissues). In the intestine, it is the “low-capacity high-affinity” SGLT1 that mediates rapid uptake of glucose and galactose. Despite its “low capacity,” uptake of large amounts of sugar is warranted by the immense expansion of the absorptive area provided by the intestinal villi and microvilli, giving rise to an SGLT1-expressing membrane surface of about 200 m^2^. Whereas d-glucose and d-galactose are absorbed in the intestine by the Na^+^/glucose cotransporter SGLT1, d-fructose is transported across the apical membrane by the facilitated fructose transporter GLUT5 (SLC2A5), followed by basolateral exit via GLUT2 (SLC2A2). Alternatively, fructose may exit via GLUT5, shown to be expressed in the basolateral membrane as well [14]. In addition to the absorptive roles of the Na^+^/glucose cotransporters, their activity also enables water absorption. This occurs through the paracellular route via solvent drag across GAP junctions. In addition, SGLT1 transporters themselves were shown to contribute to some extent toward transcellular water transport in the intestine [213]. In the kidney proximal tubules, reabsorption of about 2/3 of the filtered water occurs via the transcellular route where it is ensured by the expression of the aquaporin AQP1 in both the apical and basolateral membranes [127], while only a smaller part is absorbed through the paracellular route.

Sodium that enters the epithelial cell through Na^+^-coupled transport is pumped out of the epithelial cells into the blood via the basolateral Na^+^/K^+^-ATPase. It is this resulting transepithelial Na^+^ flux that generates the osmotic gradient necessary to drive the fluid absorption. Thus, the presence of luminal transport of glucose, galactose, and other solutes absorbed via Na^+^-coupled transport in intestine and kidney stimulates transepithelial salt and water absorption. In the intestine, crypt and villus cells cooperate during digestion to cycle fluid from the blood to the intestinal lumen and back again. Crypt cells extrude Cl^−^ through apical Cl^−^ channels into the lumen (e.g., via the cystic fibrosis transmembrane conductance regulator, CFTR), triggering release of Na^+^ and water [173]. Villus cells pump Na^+^ back into the space between cells via the coordinated action of Na^+^-coupled cotransporters and Na^+^/K^+^-ATPase. Disturbance of transepithelial glucose uptake in the intestine has significant implications in the context of fluid absorption: defects in SGLT1 in patients with the rare genetic disorder GGM (OMIM #606824) have severe diarrhea [201]. Likewise, patients with renal glucosuria (SGLT2 defect) have moderate diuresis that is partially compensated by SGLT1 in the proximal tubule S3 segments [120].

The in vivo importance of SGLT1 and GLUT2 in intestinal glucose absorption and renal reabsorption was studied in great detail using Slc5a1^−/−^ and Slc2a2^−/−^ single or double knockout mice. In addition, positron emission tomography (PET) using ^18^F-labeled glucose analogs with unique transport specificities for GLUTs and SGLTs was employed to analyze these knockout animals [149, 150]. The conclusions from these investigations are presented below. Further insights were derived from clinical data of patients with (1) GGM harboring different missense mutations in the SGLT1 gene [19, 201] (OMIM *182380), (2) GLUT2 deficiency (OMIM #227810), also known as Fanconi-Bickel syndrome (FBS), a rare disorder of glucose homeostasis that leads to accumulation of glycogen in the liver and kidney, glucose, and galactose intolerance [38, 104], and (3) familial renal glucosuria due to missense mutations in the SGLT2 gene (OMIM #233100) [109, 174]. What follows is a list of three major conclusions that can be drawn from both the studies with the transgenic mice and the clinical observations:

### SGLT1 is required for rapid glucose and galactose uptake in the intestine

This finding was revealed by the PET studies of Slc5a1^−/−^ mice [150]. Furthermore, it is consistent with the severe diarrhea in affected infants with GGM: feeding breast milk or regular infant formulas leads to life-threatening dehydration due to luminal accumulation of glucose and galactose, whereas fructose-based formulas that do not contain glucose or galactose are tolerated. Interestingly, in the absence of SGLT1, the absorption of the oral glucose load delivered to the small intestine was not completely abolished and there was slow absorption through an as yet unknown mechanism [150].

### GLUT2 is dispensable in the intestine with respect to glucose and galactose absorption but essential for glucose reabsorption in the kidney and glucose transport into and out of the liver

Evidence comes from the findings that FBS patients [109] exhibit the same phenotype as Slc2a2^−/−^ mice [149], i.e. intestinal glucose absorption in both the patients and the knockout mice was not impaired in the absence of GLUT2 [109], and that there is renal glucosuria, confirming the important role of GLUT2 in the kidney proximal tubules. How is then glucose absorbed across the intestinal basolateral membranes in the absence of GLUT2? The current notion is that basolateral glucose exit alternatively occurs via glucose phosphorylation, whereupon resulting glucose-6-phosphate is transported into the ER, followed by exit via membrane exocytosis [109]. This mechanism is analogous to the alternative pathway for glucose release from hepatocytes in the absence of GLUT2 [59]. Evidence for this stems from transepithelial glucose transport measurements of the intestine of Slc2a2^−/−^ mice [168].

Interestingly, it has recently been proposed that GLUT2 can also be targeted to the intestinal BBM where it would play an important role in intestinal sugar absorption [79], and that at very high luminal glucose concentration (e.g. 30 mM or higher), GLUT2 is recruited to the BBM, facilitating additional, SGLT1-independent apical glucose uptake [56]. However, as noted above, the subsequent studies with Slc5a1^−/−^ and Slc2a2^−/−^ mice, and the findings in patients with SLC2A2 transporter defects indicate that such a role for GLUT2 in the BBM is unlikely under normal physiological conditions [139].

### SGLT1 and SGLT2 are essential for glucose reabsorption in the kidney

The indispensability of these transporters was demonstrated by the measurement of 24 h glucose excretion in Slc5a1/Slc5a2 double knockout mice [130, 184]. In these mice, the entire filtered glucose load was excreted in the urine, while in the single SGLT2 or SGLT1 knockout mice, 67% and 98% of the filtered glucose load was reabsorbed, respectively. This is consistent with the phenotype of patients with SLC5A1 and SLC5A2 defects. Patients with defects in SGLT2 have glucosuria and excrete less than 50% of the filtered glucose load, while those with defects in SGLT1 have only mild glucosuria. Thus, in both rodents and human, the low-affinity high-capacity glucose transporter SGLT2 reabsorbs the bulk of the filtered glucose load in the proximal tubule S1 segments with GLUT2 in the basolateral membrane [207], whereas the high-affinity low-capacity glucose transporter SGLT1 reabsorbs the remaining glucose molecules from the filtrate in the late proximal tubule, with predominantly GLUT1 in the basolateral membrane [66, 76, 92, 149, 184, 207].

### SGLT2 emerges as an attractive therapeutic target for diabetes treatment

Confirmation of the physiological importance of human SGLT2 was provided by the finding that the mutations of the SGLT2 gene cause familial renal glucosuria [186]. Because the loss-of-function mutations of SGLT2 resulted in the urinary excretion of glucose, SGLT2 was confirmed to be the transporter involved in the renal reabsorption of glucose. Further validation that SGLT2 is the high-capacity transporter responsible for the reabsorption of the majority (90%) of glucose filtered at glomerulus came from the knockout mouse study in which the SGLT2 gene was disrupted [184]. The renal excretion of the glucose was much higher in SGLT2 knockout mice than that in the mice with the knockout of SGLT1 expressed in the distal straight portion (later part of S2 segments and all of S3 segment) of renal proximal tubules, which is involved in 10% of glucose reabsorption [78, 184]. The micro-puncture of the tubular fluid from the proximal tubules of knockout mice, furthermore, revealed that SGLT2 knockout almost completely abolished the glucose reabsorption in the proximal portion of proximal tubules, whereas in wild-type mice, ~ 90% of filtered glucose was reabsorbed [184]. This finally established that SGLT2 is the transporter responsible for high capacity glucose reabsorption that has been proposed to occur at the proximal portion of renal proximal tubules. These findings unmasked SGLT2 as an attractive target for the treatment of diabetic patients. Diabetes enhances renal glucose reabsorption by increasing the tubular glucose load and the expression of SGLT2, which maintains hyperglycemia. Inhibitors of SGLT2 enhance urinary glucose excretion and thereby lower blood glucose levels in type 1 and type 2 diabetes [47].

## Regulation of SGLT2 expression

It has been a long-standing question why SGLT2 does not induce a high level of functional activity when expressed in an exogenous expression systems such as X. oocytes or mammalian cultured cells, where only about a 1.5- to 3-fold increase in α-MG uptake could be achieved by expression of SGLT2, compared with control oocytes [76, 207]. This low expression level in heterologous expression systems has initially slowed down the development of specific inhibitors for SGLT2 as therapeutic lead compounds for the treatment of diabetes. Since SGLT2 has been identified as the transporter that corresponds to the previously described low-affinity/high-capacity glucose reabsorptive pathway of the renal proximal tubule S1 segments, there has been an argument that the low functional activity of SGLT2 in the exogenous expression systems contradicts its proposed role in high-capacity glucose reabsorption.

**Fig. 4:**
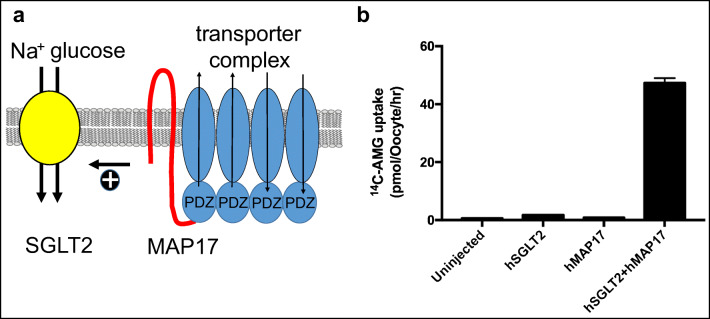
MAP17 model and augmentation of SGLT2 function. **a** Model showing MAP17 activating SGLT2 (and possibly other transporters like NHE3), via an unknown mechanism, independent of the PDZ transporter complex formation. **b** Augmentation of SGLT2 functional activity by co-expression with MAP17. The cRNAs for human SGLT2 and human MAP17 were obtained by in vitro transcription and injected individually or co-injected into X. oocytes. The uptake of ^14^C-α-MG (50 μM) was measured in ND96 buffer with 1 mM mannose 1 mM galactose [23] for 2 h and expressed as pmol/oocyte/h. The co-expression of MAP17 drastically increased SGLT2-mediated ^14^C-α-MG uptake. The experiment shown in Fig. 4b was performed by Ryuichi Ohgaki and Jin Chunhuan, Department of Bio-system Pharmacology, Graduate School of Medicine, Osaka University (unpublished data from the laboratory of Yoshikatsu Kanai)

Given that SGLT2 alone expressed in heterologous expression systems did not exhibit high levels of glucose uptake activity, unlike SGLT1 [42, 67, 76, 207], it has been proposed that for the full functional expression of SGLT2, an additional protein is required, which is not expressed in the exogenous expression systems but present in renal proximal tubules. Such a protein has indeed been successfully identified by functional expression cloning in which SGLT2, together with cRNA of clones from a cDNA library from kidney was co-expressed in X. oocytes [23]. Following screening, the protein identified was an integral membrane protein with two membrane spanning domains, designated as MAP17 (Fig. 4a). As show in the figure, it interacts with PDZK1, a scaffolding protein that was shown to interact with other membrane transporters [53]. MAP17 increased glucose uptake activity of SGLT2 in RNA-injected X. oocytes by two orders of magnitude [23] (see Fig. 4b).

A database search revealed another protein named MARDI (also called small integral membrane protein 24, SMIM24) structurally related to MAP17 [23]. Similar to MAP17, MARDI highly augmented SGLT2-mediated glucose uptake when co-expressed with SGLT2 [23]. The importance of MAP17 to maintain SGLT2 function in the renal proximal tubules under physiological condition was further confirmed in a patient of renal glucosuria, in which SGLT2 did not show any identifiable mutations. This patient was carrying a homozygous mutation in the MAP17-coding gene [23]. Among 60 individuals with familial renal glucosuria, one patient without identifiable mutations in SGLT2 displayed homozygosity to a splice mutation in the coding region of MAP17. With this finding in hand, the same research group performed an in-depth functional characterization of SGLT2/MAP17 using X. oocytes as an expression system. In agreement with previous findings, the experiments confirmed that SGLT2 is a highly selective low-affinity glucose transporter (*K*_m_ = 3.4 mM). It has a transport stoichiometry of 1:1 and shows a 10-fold higher affinity for phlorizin than SGLT1. Furthermore, electrophysiological recordings showed that SGLT2 has little pre-steady state and Na^+^-leak currents, and they described a competitive inhibitory mechanism for the SGLT2 inhibitors phlorizin and dapagliflozin [23].

Although the mechanisms through which MAP17 and MARDI enhance glucose transport activity of SGLT2 have not been fully clarified at the moment, it is intriguing that MAP17 did not change the quantity of SGLT2 protein in the exogenous expression systems [23]. Therefore, some direct or indirect effect of MAP17 on SGLT2, which is not due to membrane targeting or stabilization of the SGLT2 protein, must be involved in this high augmentation of transporter function. It was proposed that the protein conformation of SGLT2 must be somehow affected because the co-expression with MAP17 significantly increased both phlorizin binding and α-MG transport [23]. In terms of protein-protein interactions, MAP17 and MARDI are both PDZ-binding proteins with a typical PDZ-binding motif at their C-termini and that can interact with PDZ protein PDZK1 (see Fig. 4a, right part). This interaction (e.g., with other membrane transporters), however, was not essential for the stimulation of SGLT2 activity, because the deletion of the PDZ-binding motif of MAP17 did not affect MAP17’s augmentation of SGLT2 activity [23].

### Upregulation of SGLT2 expression under diabetic conditions and consequences for diabetic nephropathy

Under diabetic conditions, SGLT2 expression in proximal tubules is increased [115]. Growth and hypertrophy of the diabetic kidney may be the trigger for a general increase in the transport machinery in the proximal tubule under diabetic conditions, and it may be exacerbated with advanced nephropathy, when nephrons are lost and surviving nephrons try to compensate. Increased diabetic blood glucose levels enhance the amount of glucose filtered, provided that GFR is preserved, and renal glucose reabsorption is increased both due to the increase in glucose concentration in the glomerular filtrate and an increase of glucose transport capacity, thereby generating a substantial tubular glucose load [210]. Notably, in the early phase of diabetes, GFR is often enhanced, leading to glomerular hyperfiltration and further exacerbation of the tubular glucose load. It was also shown that maximum renal glucose reabsorptive capacity (T_m_G) is elevated in patients with type 2 diabetes (T2D) [1].

While diabetes typically elevates glomerular filtration and tubular glucose reabsorption, it additionally activates renal gluconeogenesis, thereby further exacerbating the hypoglycemia. This may be caused by (1) diabetes-related metabolic acidosis that causes renal gluconeogenesis due to the conversion of glutamine to glucose in the proximal tubule epithelial cells, with generation of ammonia and bicarbonate, to compensate the acidosis (for review see [77]), and (2) activation of the sympathetic nervous system in diabetes followed by renal stimulation of gluconeogenesis via epinephrine. All these factors give rise to a vicious circle that further nourishes hypoglycemia and hyperfiltration. The kidney’s safety feature, however, limits hyperglycemia by excretion of glucose in the urine, once blood glucose loads exceed T_m_G. Nonetheless, the diabetic kidney will undergo pathological alterations due to excessive metabolism of glucose in the tricarboxylic acid which generates enhanced oxidative stress, as well as glomerular and tubulointerstitial damage. This is followed by nephropathy with loss of nephrons, while surviving nephrons are trying to compensate.

SGLT2, which is responsible for the majority of glucose uptake in early proximal tubules, is upregulated in diabetes, but the mechanism of regulation of SGLT2 has not yet been fully established. The following pieces of information are available that help assemble the puzzle:

i. The regulation of expression of SGLT2 in T2D was investigated in Zucker rats. It was shown that SGLT1 and SGLT2 mRNA levels in Zucker diabetic obese rats were 1.6- and 4.8-fold higher than in age-matched leans, respectively [169].
ii. A similar observation was reported using human tubular epithelial cells collected from the urine of patients with T2D and maintained under cell culture conditions. The mRNA and protein levels of SGLT2 and also GLUT2 were significantly increased in the epithelial cells obtained from diabetic patients compared with those from normal subjects [135]. Accordingly, sugar uptake was increased in the cells from diabetic patients.
iii. The high level of glucose itself did not affect SGLT2 expression in tubular cells but insulin significantly increased tubular SGLT2 levels through the generation of oxidative stress [110]. Because hyperinsulinemia is accompanied with the onset and early stage of T2D [80], the upregulation of SGLT2 at least at the early stage of T2D thus could be due to the stimulation by insulin. Indeed, increased glucose absorption in proximal tubule membrane vesicles of hypertensive rats was found to be due to induction of SGLT2 expression leading to kidney damage due to the generation of reactive oxygen species (ROS) [137]. Increased SGLT2 expression in the diabetic condition may lead to kidney damage via the production of reactive oxygen species.
iv. HNF1α was shown to control renal glucose reabsorption in mouse and human. It was demonstrated that HNF1α controls SGLT2 expression by direct transcriptional activation, whereas SGLT1 and GLUT2 were not affected [129]. In addition, it was shown that renal proximal tubular reabsorption of glucose was reduced in patients with maturity onset diabetes of the young type 3 (MODY3) that is caused by mutations of the HNF1α gene.
v. In addition, it was shown, based on studies with db/db mice with diabetes and high-glucose-cultured porcine PT LLC-PK1 cells in a two-chamber system treated with the SGLT2 inhibitor canagliflozin, that SGLT2 expression was stimulated by basolateral high glucose concentrations through activation of the so-called GLUT2/importin-α1/HNF-1α pathway, while the expression of the NAD^+^-dependent protein deacetylase SIRT1 decreases, leading to a deficiency of autophagy [179]. Upregulation of SGLT2 expression in diabetes may be caused through sensing of basolateral hyperglycemia via GLUT2 [111, 119]. Interestingly, SGLT2 inhibitors induce SIRT1, together with adenosine monophosphate-activated protein kinase AMPK, which have been shown to stimulate autophagy, thereby ameliorating cellular stress and glomerular and tubular injury [121].
vi. It was reported that mTOR signaling is involved in the upregulation of the expression of nutrient transporters, including SGLT2, in renal proximal tubule cells. The combination of the deletions of the mTOR complex 1 (mTORC1) and 2 (mTORC2) by the conditional knockout of the regulatory associated protein of mammalian target of rapamycin (RAPTOR), which is an essential component of mTORC1, and the rapamycin-insensitive companion of mammalian target of rapamycin (RICTOR), which is essential for mTORC2, resulted in the development of a Fanconi-like syndrome involving glucosuria in mice [57]. Interestingly, proteomics and phosphoproteomics of freshly isolated kidney cortex identified either reduced expression or loss of phosphorylation at critical residues of nutrient transporters, which leads to reduced nutrient transport due to perturbation of the endocytotic machinery. For example, phosphorylation of mouse Sglt2 at S623 was reduced in kidney cortex by the loss of mTORC1 [57]. This phosphosite is highly conserved between species, including human SGLT2 (S624) [50]. It was shown that the phosphorylation at this site increases membrane insertion of SGLT2 and enhances glucose transport [50]. The loss of phosphorylation at S623 was, thus, proposed to explain the glucosuria caused by the mTORC1/mTORC2 deletion.
vii. It remains to be determined, whether the scaffolding protein MAP17 is involved in the functional upregulation of proximal tubule transporters such as SGLT2 and also NHE3 (SLC9A3). Both of these transporters are stimulated by insulin to enhance Na^+^ and glucose reabsorption [83, 110], but a role of MAP17 in the regulation of SGLT2 and other relevant transporters in the diabetic conditions has not been reported thus far.

In conclusion, further studies will still be needed to fully understand the mechanisms of upregulation of both SGLT2 and GLUT2 in the different diabetic conditions.

## Regulation of SGLT1 in the kidney in the absence of SGLT2

SGLT2 inhibition shifts glucose uptake further downstream toward S3 segments and thick ascending limbs (TAL). In the kidney, SGLT2 and SGLT1 account for at least 90% and about 3% of fractional glucose reabsorption (FGR), respectively, while euglycemic individuals treated with an SGLT2 inhibitor maintain an FGR of 40–50% [138]. This value is similar to the values of Sglt2 knockout mice [130]. The increase of the contribution of SGLT1 toward glucose reabsorption following SGLT2 inhibition was studied in mice [138]. Selective SGLT2 inhibition in mice revealed that SGLT2 and SGLT1 account for renal glucose reabsorption in euglycemia, with 97 and 3% being reabsorbed by SGLT2 and SGLT1, respectively. However, when SGLT2 is fully inhibited by an SGLT2-inhibitor, there is an increase in SGLT1-mediated glucose reabsorption which explains why only 50–60% of filtered glucose gets excreted [138]. Thus, when SGLT2 function is lacking, either due to SGLT2 inhibition or lack of gene expression in Sglt2−/− mice or in patients with familial glucosuria, there is significant compensation by SGLT1. An olfactory G protein-coupled receptor, Olfr1393, expressed in the kidney proximal tubule was proposed to serve as a physiological regulator of SGLT1 expression [138]. Alternatively, the G protein-coupled glucose-sensing receptor T1R3 that was originally identified in sheep and rodent intestine [106, 158] might also be a candidate for SGLT1 regulation in kidney proximal tubules as well [215]. However, the precise mechanism by which renal SGLT1 is upregulated to compensate for the lack of SGLT2 function still awaits further investigation.

## Structural biology; molecular modeling; SGLT1 and SGLT2; molecular architecture

Proteins of the SSS family usually contain 10–14 transmembrane helices (TMHs), and the tertiary structures of two representative members are known. The sodium/galactose symporter from *Vibrio parahaemolyticus* (vSGLT) was crystallized with bound galactose in an inward-open conformation (PDB ID: 3DH4) [40]. The structure of the inactive mutant variant K294A has also been solved and displays a very similar inward-open conformation in the absence of the bound sugar (PDB ID: 2XQ2) [194]. In addition, another protein from the same family, the sialic acid transporter SiaT from *Proteus mirabilis*, was crystallized in the presence of sialic acid (*N*-acetylneuraminic acid) (PDB ID: 5NV9) [192]. Interestingly, this structure features the protein in an outward-open conformation.

The above structures show that SSS transporters share the same structural fold as APC (amino acid-polyamine-organocation) transporters, featuring a transporter core formed by 5 + 5 TMHs in an inverted repeat arrangement. Strikingly, a bound Na^+^ ion was also observed in the structures of vSGLT and SiaT in a location analogous to the “Na2” cation-binding site present in many APC-fold transporters [25, 125, 126, 161, 204], indicating the conservedness of the Na2 site across the structural superfamily. Many APC-type transporters with a 2:1 cation:solute transport stoichiometry also feature a second cation-binding site (termed “Na1”), which is typically closer to the substrate-binding pocket. However, the SiaT structure suggests that a second Na^+^ ion binds to a distinct (third) site, termed “Na3,” implying that the Na1 site found in other APC-type transporters is not conserved in the SSS family [192]. This is also likely to be the case for human SLC5A1.

The mechanism of transport has been extensively studied for human SLC5A1 using a variety of methods (Fig. 2). Experimental data have long supported a transport model where Na^+^ ions bind to the protein first, followed by glucose [122]. Initial binding of Na^+^ is expected to occur to the Na2 site (termed “Na^+^_2_” in Fig. 2), then quickly jump to the Na3 site (“Na^+^_1_” in Fig. 2) [192]. After the second Na^+^ binding to “Na^+^_2_” (Fig. 2), the two bound Na^+^ ions are expected to stabilize the transporter in an outward-open state and increase affinity for sugar binding [40, 148]. Binding of sugar induces the formation of the extracellular gate (Y87, F424, M73 in vSGLT; F101, F453, L87 in hSLC5A1; F98, F453, L84 in hSLC5A2, respectively) and thus extracellular closure [40]. The subsequent transition of the protein to an inward-facing state destabilizes the Na^+^ bound at the “Na^+^_2_” site, and this ion is proposed to be released on a short time scale [194]. These rearrangements are also expected to disrupt the “Na^+^_1_” site and cause the dissociation of Na^+^ bound at “Na^+^_1_” (Fig. 2). The release of Na^+^ ions allows Y263 in vSGLT (Y290 in hSLC5A1 and hSLC5A2), which was shown to be in a stacking interaction with the bound sugar ring and was proposed to be an intracellular gate residue, to adopt a different conformation and thus facilitate the dissociation of the bound sugar [194]. Interestingly, further intracellular gating residues R260 and D182 in SiaT, where D182 also forms part of the “Na^+^_1_” (Na3) binding site, are also conserved in both human SLC5A1 and SLC5A2 (R300 and D204/D201, respectively).

In the inward-open holo structure of vSGLT, the hydroxyl groups of the bound galactose molecule make extensive hydrogen-bond contacts with protein residues, and its heterocyclic carbon ring engages in a stacking interaction with aromatic side-chains of the protein. To assess the structural details of sugar binding to human SLC5A2 during the early steps of the transport cycle, we explored the binding modes of glucose to an outward-open state of human SLC5A2 using a combination of homology-based modeling and molecular docking (Hediger et al., unpublished observation). To this end, we have used MODELLER 9.23 [195, 196] to build structural models of human SLC5A2 based on the structure of SiaT (PDB ID: 5NV9) using a manually adjusted sequence alignment more suitable for loop modeling. To account for conformational variation, we have generated a total of 1000 protein models. We have performed molecular docking of *α*-d-glucose on all 1000 protein models using AutoDock 4.2.6 [108] with flexible ligand and rigid protein side-chains. The search space was defined as a cube of 22.5 × 22.5 × 22.5 Å centered on the location of the galactose ring from the vSGLT structure (PDB ID: 3DH4) after fitting to SiaT. For each model of SLC5A2, 10 docked poses of glucose were generated. The best 500 of the resulting 10′000 glucose poses were energy minimized using SMINA [86] while keeping neighboring protein side-chains flexible, and the resulting ligand poses rescored using the Vinardo scoring function [134]. From the resulting set, glucose poses where the distance of the center of the docked glucose and the original galactose ring from vSGLT was less than 2 Å were ranked according to their calculated binding energies. The glucose pose with the lowest energy yielded by the above described process is shown in Fig. 6a. Importantly, the plane of the glucose ring predicted in our study is parallel to that of galactose observed in the vSGLT structure, while all hydroxyl groups of the sugar ligand are in hydrogen-bonding orientation with protein residues. This is in contrast to a recent analogous study [13], where the sugar was predicted to bind in a perpendicular orientation. Based on our model, SLC5A2 residues K321, E99, S287, and N75 directly coordinate the sugar ligand, whereas the stacking interaction with Y290 expected in analogy to vSGLT is absent due to steric reasons. The formation of this interaction might be a major driving force for the outward-to-inward transition of the apo transporter. Importantly, our results, also in-line with previous studies [13], explain the deleteriousness of the naturally occurring loss-of-function variant K321R associated with familial renal glucosuria [103]. However, other mutations near the sugar-binding site, such as F98L [208, 209], A102V [18], and G77R [193], might also cause loss-of-function in hSLC5A2 through directly interfering with glucose binding.

Interestingly, during our docking studies, we have consistently observed docked glucose poses where the pyranose ring center was ~ 7 Å away from its expected location according to the vSGLT structure (Fig. 6f). Structural analysis of the protein environment near this region suggests a plausible sugar-binding site, where glucose would form hydrogen-bonds with protein residues D158, K154, Y150, Y290, and the backbone amino and carbonyl groups of G77 and N75, respectively. Remarkably, D158 is conserved in all 7 sugar-transporting human SLC5 proteins, but not in non-sugar transporting SLC5 members, while the other 3 residues are partially conserved (K154 not in hSLC5A4, Y150 not in hSLC5A4 and hSLC5A10, Y290 not in hSLC5A9). We believe that these residues might form an intermediate sugar-binding site during the sugar translocation process, but further investigation is needed to clarify their roles in the transport process.

## Development of inhibitors, analysis, and inhibition kinetics of canagliflozin acting on SGLT2 and SGLT1

The observation that the suppression of renal glucose reabsorption ameliorates diabetic conditions was first reported in 1987. To demonstrate the contribution of glucotoxicity to the development and exacerbation of the diabetic condition, phlorizin was administered to partially pancreatectomized diabetic rats with continuous subcutaneous infusion through small implantable minipumps. In this study, it was demonstrated that phlorizin, by increasing urinary glucose excretion, normalized plasma glucose levels and completely corrected abnormalities associated with the diabetic conditions such as insulin deficiency and hyperglycemia [142]. Furthermore, the correction of hyperglycemia with phlorizin treatment normalized tissue sensitivity to insulin in the diabetic rats [142]. In this study, phlorizin was used to normalize the blood glucose level without mediating insulin actions to demonstrate the contribution of glucotoxicity to diabetic conditions [142]. It was shown that phlorizin recovered the function of β-cells secreting insulin and ameliorated tissue insulin resistance. Although phlorizin is a high-affinity inhibitor of Na^+^/glucose transporters, it has previously not been clinically developed as an anti-diabetic drug. One of the main reasons was that phlorizin is not orally administrable. Phlorizin is an *O*-glycoside and, therefore, it is hydrolyzed in the intestinal lumen by β-glucosidase and broken down into glucose and phloretin that inhibits facilitative glucose transporters (GLUTs) [36]. The second reason was that phlorizin is less selective. Phlorizin, in fact, inhibits both SGLT1 and SGLT2 [42]. When phlorizin is orally administered, the strong inhibition of intestinal SGLT1 would cause severe life-threatening diarrhea similar to the glucose-galactose malabsorption caused by the loss-of-function mutations of the SGLT1 gene [178].

To overcome such disadvantages of phlorizin, its derivative T-1095 was synthesized [117]. T-1095 is an orally administrable esterified prodrug that is absorbed from the gastrointestinal tract and converted to an active form by the esterase in the liver [117]. Because it is inactive in the lumen of the small intestine, T-1095 does not inhibit SGLT1 in the small intestine [4]. Although the SGLT2/SGLT1 selectivity of the active form of T-1095 is much less than that of SGLT2 inhibitors currently in clinical use, orally administered T-1095 could selectively inhibit renal glucose reabsorption while minimally affecting intestinal glucose absorption [117]. In the pre-clinical studies, orally administered T-1095 increased glucose excretion into urine in diabetic animals and successfully decreased both blood glucose and HbA1c levels [117]. It suppressed the postprandial hyperglycemia after a meal load in diabetic animals. The hypertriglyceridemia and the development of microalbuminuria associated with diabetic conditions were also ameliorated [4, 117]. The results obtained from T-1095 on diabetic animal models confirmed that the inhibition of renal glucose reabsorption could be a novel therapeutic approach for diabetes mellitus.

**Fig. 5:**
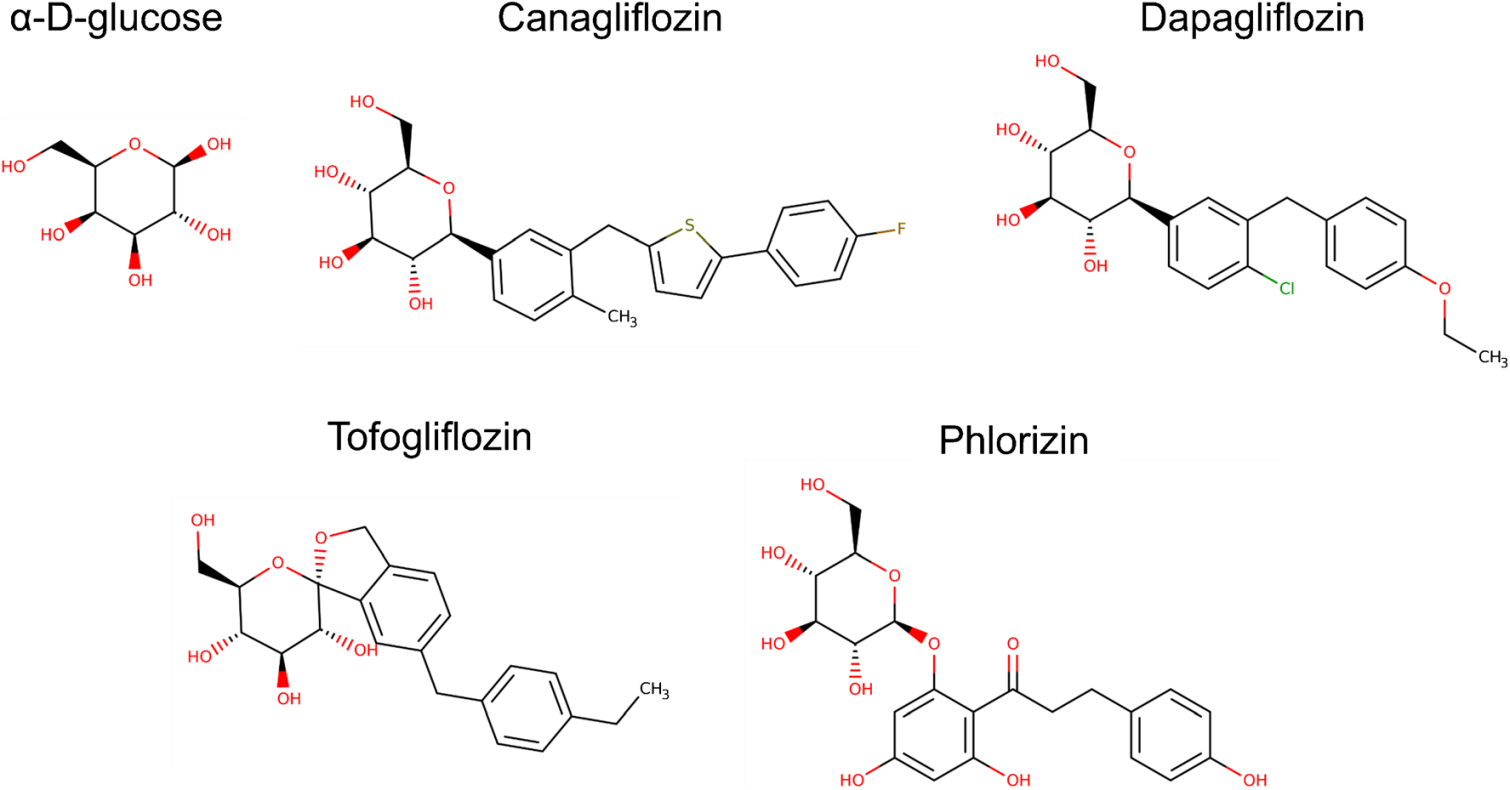
Chemical structures of various inhibitors of the SLC5 proteins in comparison with *α*-d-glucose. While phlorizin is a relatively non-selective inhibitor of sodium-coupled sugar transporters, canagliflozin, dapagliflozin, empagliflozin, ipragliflozin, luseogliflozin, and tofogliflozin are selective (250-fold, 1200-fold, 2500-fold, 360-fold, 1650-fold, 2900-fold, respectively) inhibitors of human SLC5A2 vs human SLC5A1 (see text for details)

The SGLT2 inhibitors currently in clinical use include dapagliflozin, canagliflozin, empagliflozin, ipragliflozin, tofogliflozin, luseogliflozin, and ertugliflozin [2, 89, 113] (see Fig. 5). Sotagliflozin has been abandoned while enrolling into large clinical heart and kidney outcome trials. Although these compounds have been synthesized based on the phlorizin structure, they are *C*-glycosides, distinct from phlorizin that is an *O*-glycoside, making sure that they are not hydrolyzed in intestine and metabolically stable in blood after absorption from intestine [6]. Therefore, they are administered once a day orally for the treatment of patients with diabetes. The additional advantage of these clinically used SGLT2 inhibitors is their high-affinity and selectivity for SGLT2 except sotagliflozin designed as a dual inhibitor, inhibiting both SGLT2 and SGLT1. They are 150 to ~ 3000 times more selective for SGLT2 than SGLT1 except sotagliflozin [89].

### Physiological basis of clinical benefits of SGLT2 inhibitors

SGLT2 inhibitors came in use clinically in 2012, and a bunch of clinical evidence had to be brought together to demonstrate their effectiveness in the treatment of diabetic patients. SGLT2 inhibitors reduce blood glucose and HbA1c, recover β-cell function, and ameliorate insulin resistance. They, furthermore, decrease body weight and blood pressure, and lower blood urate and triglyceride levels [6]. Large-scale clinical trials have also demonstrated that they are effective in the suppression of cardiovascular events and renal complications [212]. The usefulness of SGLT2-selective inhibition in the treatment of diabetes was also confirmed in Slc5a2 knockout mice showing that SGLT2 deletion reduced hyperglycemia associated with high-fat diet and obesity, improved glucose intolerance, and increased glucose-stimulated insulin secretion [74]. Not only the effectiveness but also the clinical safety of the drugs was established. It is also well recognized that the risk of hypoglycemia is relatively low when using SGLT2 inhibitors [6].

### Effects of SGLT2 inhibitors on extra-renal SGLT2

The expression of SGLT2 was originally proposed to be kidney-specific, but detailed expression studies have revealed extra-renal expression of SGLT2. In pancreatic α-cells secreting glucagon, SGLT2 was shown to be expressed with SGLT1 [15]. It was demonstrated that the SGLT2 inhibitor dapagliflozin triggers glucagon secretion in human islets by directly acting on islet α-cells. This may explain why SGLT2 inhibitors cause the paradoxical increase of plasma glucagon levels [20].

The other example of extra-renal expression of SGLT2 is in cancers. It has been demonstrated that cancer cells upregulate glucose transporters such as GLUT1 to compensate increased demand of glucose associated with the Warburg effect [71]. SGLT2 was also shown to be expressed in some types of cancers such as pancreatic and prostate cancers, together with SGLT1 [81, 153]. It is intriguing that SGLT2 inhibitors reduce tumor growth and survival in a xenograft model of pancreatic cancer [153].

### Molecular docking of the gliflozin inhibitors of SGLT2

**Fig. 6:**
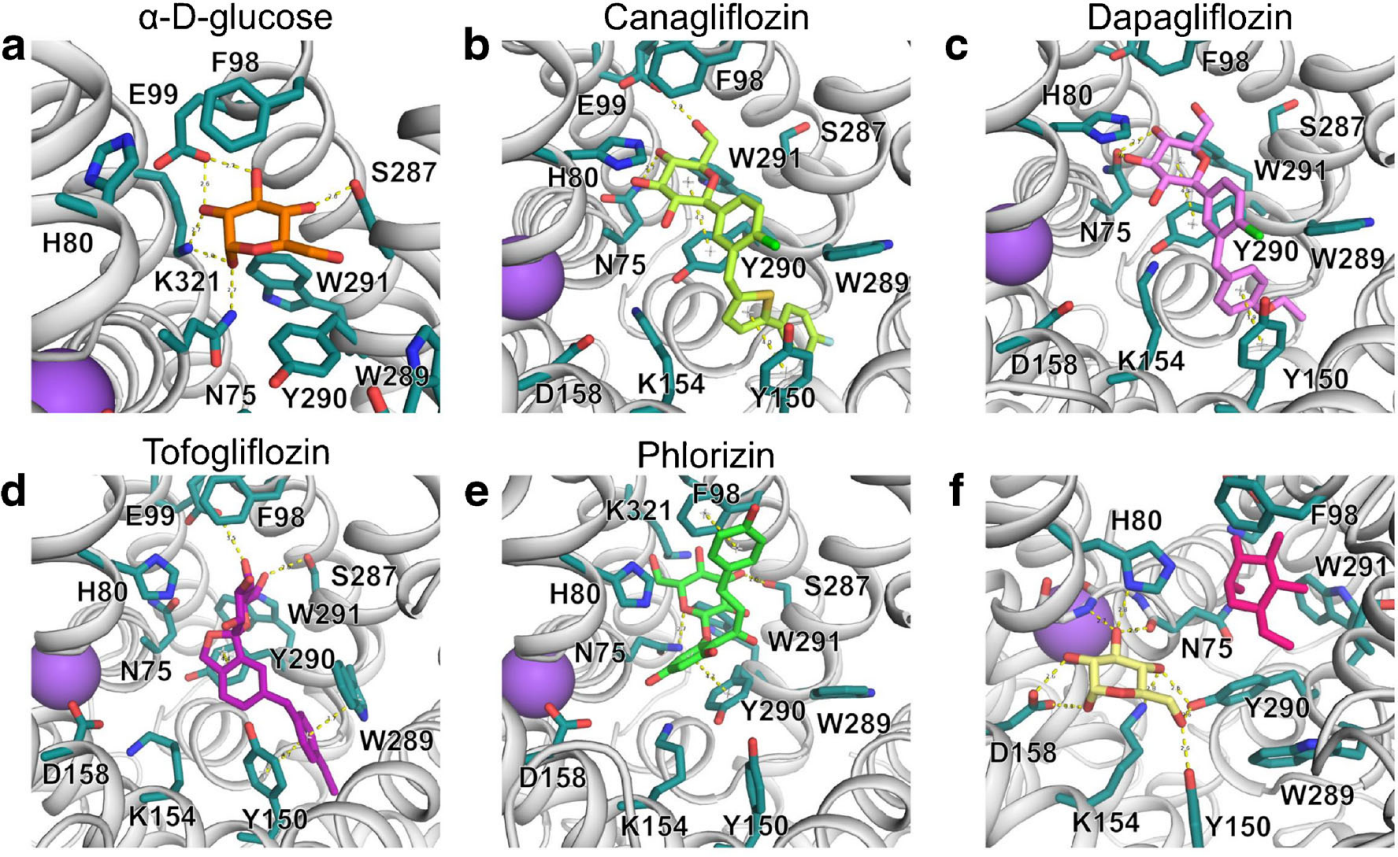
Binding modes of glucose and various inhibitors to human SGLT2 (SLC5A2). The protein backbone is shown as gray ribbon. Selected individual amino acid side-chains are shown in dark cyan. Purple sphere represents the Na^+^ ion. Hydrogen bonds and aromatic ring stacking interactions are shown as dashed lines. Docked ligands are shown in various colors (**a**–**e**). Panel **f** shows a putative alternative sugar-binding site of docked *α*-d-glucose (yellow) compared with the canonical binding pose (pink) (see text for details)

To elucidate the binding of gliflozins to human SLC5A1 and SLC5A2 proteins, we have applied our computational docking protocol described above to the generation of docked poses of phlorizin, canagliflozin, dapagliflozin, and tofogliflozin. The chemical structures of these compounds are presented in Fig. 5. Based on the assumption that the sugar moiety of these compounds should occupy the same binding site as glucose, we have employed filtering of the resulting poses based on the distance of the center of their pyranose rings to that of galactose in the vSGLT structure, similarly as we did for glucose docking. The best-scoring (the lowest binding energy) of the resulting poses is shown in Figs. 6a–e (Hediger et al., unpublished observation). Interestingly, the sugar moieties of the docked inhibitors are all rotated compared with the docked glucose molecule (Fig. 6a), but their rings still share a plane parallel to the membrane bilayer, similar to glucose. A notable exception is tofogliflozin, where the sugar moiety is sterically fixed compared with the aglycon tail and was predicted to bind in a perpendicular orientation. For phlorizin, which is a less selective inhibitor of both hSLC5A1 and hSLC5A2, aromatic stacking interactions are noticeable between the aglycon tail and protein residues Y290 and F98. This aligns well with experimental results showing that F101 in human SLC5A1 (analogous to F98 in human SLC5A2) is important for phlorizin binding [148]. For the other 3 inhibitors, their aglycon tails reach into a binding pocket lined by protein residues Y150 and W289, which engage in aromatic stacking interactions with the ligands. Notably, this binding pocket is not utilized by phlorizin and its existence might be responsible for the significantly higher selectivity of the other 3 inhibitors for hSLC5A2. While no obvious differences in sequence can be observed in this binding pocket between hSLC5A1 and hSLC5A2, dynamic factors such as extracellular gate opening could affect the accessibility or the size of this binding pocket. Notably, one relevant sequence difference is in the hinge region of the extracellular gate, where A464 in hSLC5A2 vs G464 in hSLC5A1 could provide more rigidity and thus a more well-formed binding pocket in the case of human SLC5A2 compared with human SLC5A1. Another analogous study on the selectivity of gliflozin compounds has suggested that these highly selective compounds interact with the C-terminal region of extracellular loop 5 (EL5) directly through residue H268, and that their binding leads to a partial closure of the extracellular gate [13]. The authors argue that direct interaction with the non-conserved residue H268 in hSLC5A2 (D268 in hSLC5A1) contributes to the selectivity of gliflozins, augmented by the absence of the second bound Na^+^ ion at the Na3 site in hSLC5A2, which shifts the conformational equilibrium of the transporter to favor extracellular gate closure [13]. In summary, dynamic effects are likely to be responsible for the selectivity gliflozin compounds, and further crystallographic studies are necessary to verify the precise binding modes of these inhibitors to human SLC5A1 and SLC5A2.

## Clinical benefits of the SGLT2 inhibitors in type 1 and type 2 diabetes and role of the renin-angiotensin system

SGLT2 inhibitors are effective glucose-lowering drugs that primarily act by blocking the SGLT2 transporter in the renal proximal tubule. The specific clinical benefits of the drugs are as follows.

### Body weight reducing effects of SGLT2 inhibitors

These drugs lower body weight in T2D already within a few weeks after starting the therapy [93]. Initially, the weight loss is mostly due to diuretic effect through increased osmotic diuresis and a related decrease of the extracellular volume [205]. However, the increased urine volume of patients was normalized again after 4 weeks, which highlights that there is a homeostatic adaptive mechanism [3]. This weight reduction is accompanied by reduction of total body fat percentage due to the negative energy balance, increased lipolysis, and fatty acid oxidation, with simultaneous inhibition of lipogenesis and increased formation of ketone bodies. [45, 131]. The lean tissue mass that mainly includes the muscle mass showed no significant change following SGLT2 inhibitor treatment in one study [157], while in another study, the loss of muscle mass, alongside the loss of fat mass in patients with T2D, was observed in response to SGLT2 inhibition [82].

### Nephroprotective effects

Elevation in glomerular filtration rate (GFR) is observed early in the pathogenesis of T1D and T2D. At the single-nephron level, diabetes-related renal hemodynamic adaptations occur to counteract loss-of-functional nephron mass, thereby increasing glomerular hydraulic pressure, a phenomenon known as glomerular hyperfiltration, leading to irreversible nephron damage and contributing to initiation and progression of kidney disease in diabetes. Reduced GFR is one of the key markers of predicting the risk of end-stage renal disease and renal death in diabetes. Reported prevalence of hyperfiltration at the whole-kidney level is 10–67% in T1D and 6–73% in patients with T2D [175]. Hyperfiltration in T1D is thought to precede the onset of albuminuria and decline in renal function and to predispose to progressive nephron damage by increasing glomerular hydraulic pressure and transcapillary convective flux of the ultrafiltrate carrying macromolecules including albumin. Increased GFR in single remnant nephrons to compensate for reduced nephron numbers further accelerates renal damage in diabetes. SGLT2 inhibitors have general nephroprotective effects. As outlined below, in diabetes patients, SGLT2 inhibitors activate the tubuloglomerular feedback (TGF) mechanism with the inhibition of glomerular hyperfiltration, which is characteristic in diabetes patients. Beneficial effects are also anticipated for patient groups without diabetes, such as chronic kidney disease (CKD) patients, but confirmatory studies are still required.

### Inhibition of glomerular hyperfiltration by SGLT2 inhibitors in patients with T1D via the tubuloglomerular feedback mechanism

As already noted, glomerular hyperfiltration is a recognized risk factor for the development and progression of diabetic kidney disease (DKD). Also, it is a risk factor in the development of chronic kidney disease in general. Hyperfiltration of the remaining nephrons is due to activation of the renin-angiotensin-aldosterone system (RAAS), leading to increased systemic blood pressure, increased angiotensin sensitivity of efferent arterioles and to tubuloglomerular feedback (TGF) adaptation in the afferent arterioles.

In addition, Na^+^ reabsorption in the proximal tubules is increased in T1D and in T2D due to chronic hyperglycemia. As already noted, this results in the upregulation of the expression of both SGLT2 and GLUT2 [115, 135]. Thereby, Na^+^ (i.e. NaCl) exposure at the macula densa of the juxtaglomerular apparatus diminishes which, due to a “renal misinterpretation” of a “reduced effective arterial blood volume”, leads to an increased dilation of the afferent arteriole, in order to preserve the alleged low intraglomerular pressure and the GFR in the spirit of a correct autoregulation. This resulting unfavorable hyperfiltration further accelerates nephron destruction and progression of renal failure. Clinical studies using the SGLT2 inhibitor empagliflozin revealed that SGLT2 inhibition significantly reduces the hyperfiltration in patients with T1D mellitus [22]. A recent study provided the first direct demonstration of changes in renal hemodynamic function by SGLT2 inhibitors using in vivo glomerular visualization with multi-photon microscopy in a diabetic animal model [82]. Within 2 h after application a single dose of empagliflozin, they observed an acute drop in hyperfiltration and albumin excretion in the diabetic mice. At the same time, the investigators visualized a distinct reduction of the extended diameters of the afferent glomerular arterioles. In parallel, the amount of the excreted adenosine in the urine significantly increased. The effect of the SGLT2 inhibitor could be prevented when using a selective adenosine blocker, thereby validating the hypothesis that SGLT2 inhibition leads to increased adenosine production at the macula densa which ultimately leads to constriction of the afferent arterioles.

**Fig. 7:**
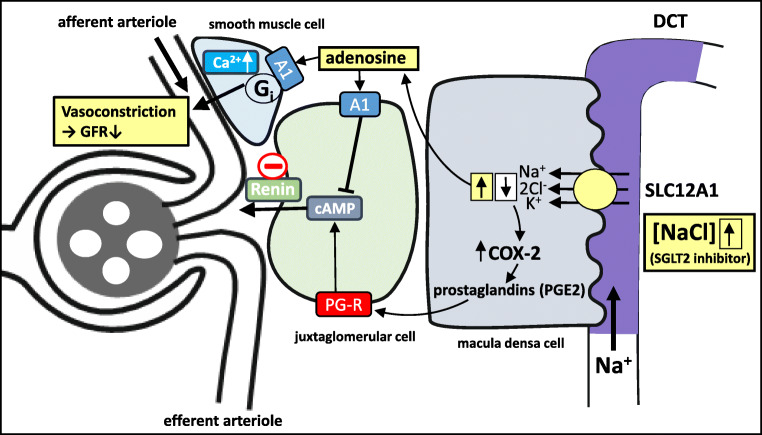
Effect of SGLT2 inhibition on renal hemodynamics via the tubuloglomerular feedback (TGF) mechanism: the macula densa cells are specialized epithelial cells that form the macula densa as part of the distal tubule sensing system of the same nephron. As shown on the right, these cells detect the luminal NaCl concentration in the tubular fluid. NaCl detection occurs after its uptake by SLC12A1 (NKCC2). Elevated filtration at the glomerulus or reduced reabsorption of Na^+^ and water in the proximal tubule causes the tubular fluid at the macula densa to have a higher concentration of luminal NaCl. Inhibition of SGLT2-mediated Na^+^-coupled glucose transport significantly increases NaCl exposure at the macula densa. This is followed by increased transport activity of SLC12A1 (NKCC2) in macula densa cells. As part of the sensing mechanism, this ultimately leads to extracellular accumulation of adenosine in the juxtaglomerular interstitial space (see text for details). Adenosine can then directly act on G_i_-coupled A1 receptors expressed on afferent arteriolar smooth muscle cells [63, 87], triggering vasoconstriction of the afferent arterioles to reduce GFR, as shown in Fig. 8a and b

The mechanism that leads to the constriction of the afferent glomerular arterioles via the tubuloglomerular feedback (TGF) to counteract the hyperfiltration in the diabetic condition is outlined in Fig. 7. In addition, Box 1 presents the role of macula densa cells in controlling GFR and renin release via TGF. As shown in Fig. 7, in T1D, inhibition of the SGLT2-mediated Na^+^-coupled glucose reabsorption in the proximal tubules results in an increase in the luminal NaCl concentration at the macula densa above the threshold value. As a result, macula densa cells trigger the reduction of renin release to suppress the renin-angiotensin-aldosterone system (RAAS) and vasoconstriction of the afferent arterioles to reduce hyperfiltration that is commonly associated with diabetes. This is achieved via generation of extracellular adenosine in the juxtaglomerular interstitium (see Box 1). As shown in Fig. 7, adenosine can directly act on G_i_-coupled A1 receptors expressed on afferent arteriolar smooth muscle cells [63, 87] to trigger vasoconstriction of the afferent arterioles to reduce GFR. In addition, adenosine causes via A1R inhibition of renin release in juxtaglomerular cells (see Fig. 7). The latter is believed to occur via A1R-triggered TRPC6 channel-mediated calcium entry, followed by calcium-mediated inhibition of renin release [63]. Thus, adenosine signaling through the TGF is of central importance in preventing diabetic hyperfiltration via SGLT2 inhibition, leading to the nephroprotective action of SGLT2 inhibitors, with prevention of subsequent damage of remaining nephron segments. The mechanism explains why in T1D, after SGLT2 inhibition, there is an initial decrease in GFR that is independent of systemic blood pressure.

Box 1 Role of macula densa cells in TGF-mediated control of GFR and renin secretion.

**Table:** 

**Fig. 8:**
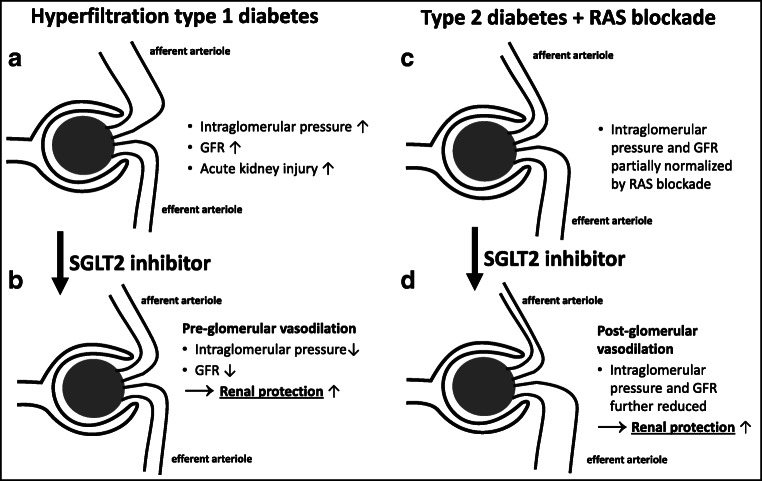
Hypothesized renal hemodynamic effects of SGLT2 inhibitors: **a** and **b** beneficial effects in type 1 diabetes patients; **c** and **d** beneficial effects in type 2 diabetes patients treated with a renin-angiotensin system (RAS) blocker

Fluid flow through the kidney nephron is being kept within a narrow range under healthy conditions for optimal maintenance of salt and water balance. The macula densa cells control the tubuloglomerular feedback (TGF) to fine-tune GFR [156]. As shown in Fig. 7, when the luminal NaCl concentration at the macula densa rises, as during ECV expansion or in response to SGLT2 inhibition, NaCl entry into macula densa cells via NKCC2 leads to the production of adenosine. The macula densa cells do not have enough Na^+^/K^+^-ATPases on their basolateral surface to excrete the added Na^+^ taken up. This results in osmotic swelling and the current concept is that the swelling leads to exit of ATP across the basolateral membrane via stretch-activated, non-selective maxi-anion channels, followed by its conversion to adenosine via the ecto-5′-nucleotidase channels [16, 87, 143, 144]. Adenosine then interacts with adenosine 1 receptors (A1R) on vascular smooth muscle, resulting in contraction of afferent arterioles and reduction of GFR (the TGF response). On the other hand, in response to decreased luminal NaCl concentration at the macula densa (the reverse situation illustrated in Fig. 8), i.e., in the context of reduced blood pressure or glomerular filtration, NKCC2 transport activity is reduced, which causes release of prostaglandin in macula densa that activates signaling cascades to promote renin release and activation of the renin-angiotensin-aldosterone system (RAAS). This in turn increases the blood pressure via aldosterone by increasing renal reabsorption of sodium and water. In addition, with luminal NaCl concentrations below the threshold value, vasodilation of afferent arterioles occurs, which increase glomerular filtration pressure and tubular fluid flow.	

Recently, it was observed that macula densa cells of mouse and human kidney also express SGLT1 where it is anticipated to serve as a glucose sensor to further regulate TGF, whereby glucose taken up mediates upregulation of nitric oxide (NO) synthase NOS1, followed by generation of NO that will decrease TGF and promote glomerular hyperfiltration [215]. In support of this concept, another study revealed that (1) deletion of SGLT1 reduced glomerular hyperfiltration in diabetic Akita mice but did not markedly change blood glucose levels and (2) the increase in macula densa NOS1 expression observed in diabetic Akita mice was abolished in the absence of SGLT1 [164]. Future studies on this interesting topic will be of great interest. Additionally, further studies on the localization of SGLT1 in distal tubules would be important, because earlier reports of rat [76, 92, 207] and human kidney [191] were not able to provide evidence for SGLT1 expression in macula densa cells. It will also be of interest to know whether SGLT1 is upregulated in macula densa cells in response to SGLT2 inhibition, in analogy to SGLT1 in the proximal straight tubules.

### Beneficial effects of SGLT2 inhibitors in T2D-based and combination therapies

Glomerular hyperfiltration with elevated GFR is an important risk factor for the development of diabetic kidney disease (DKD). This is also true for T2D patients, although hyperfiltration is more difficult to diagnose, because they often have a normal or even a reduced GFR due to loss of nephrons. It is likely, however, that they still exhibit hyperfiltration at the single nephron level. Blockage of the renin-angiotensin system (RAS) by antihypertensive agents such as ARBs (angiotensin receptor blockers) has proven effective for the treatment of T2D patients. ARBs improve DKD progression by lowering glomerular pressure and hyperfiltration, mainly by dilating efferent arterioles due to lack of constriction [85]. In T1D, adenosine plays a central role in executing the nephroprotective response of SGLT2 inhibitors, by ameliorating preglomerular arteriolar dilation and hyperfiltration via the adenosine receptor A1R, acting on the vasoconstriction of afferent arterioles. A recent study was launched to examine the renoprotective benefits of a combination therapy with the SGLT2 inhibitor dapagliflozin in T2D patients treated with ARBs [185]. The study was undertaken because (1) it was unclear how exactly SGLT2 inhibitors affect renal hemodynamics in patients with T2D whose renal physiology differs from that of previously studied T1D patients with hyperfiltration and (2) it was unknown whether the effects of SGLT2 inhibitors are diminished in response to glucose lowering. Therefore, the effects of dapagliflozin on renal hemodynamics in T2D patients treated with the blood sugar lowering agent metformin, together with ARB, were examined as well. The study revealed that the beneficial renal hemodynamic effects of SGLT2 inhibitors are fully independent of the glucose-lowering effects. Interestingly, lowering GFR was accompanied by a stable or even lowered renal vascular resistance (RVR), suggesting that the acute decline in GFR is mainly caused by post-glomerular vasodilation, rather than preglomerular restriction. Interestingly, in this study, the concentration of adenosine, which plays a key role in mediating the positive effects of SGLT2 inhibitors, was also significantly increased in T2D patients. Compared with the hyperfiltration in patients with T1D, however, baseline GFR and effective renal plasma flow (ERPF) were much lower in the T2D study, indicating that the preglomerular arteriolar diameter was already narrow, thus limiting or prohibiting further preglomerular vasoconstriction by adenosine (see Fig. 8c and d). Adenosine may, however, induce further post-glomerular vasodilation via A2R instead of preglomerular vasocontraction (in accordance with the concept that activation of adenosine receptors of type A1 leads to constriction of afferent arterioles and that of type A2 to dilation of post-glomerular arteries) [183]. Therefore, the beneficial effect of SGLT2 inhibitors on the renal hemodynamics of these T2D patients with RAS blockade is most likely due to enhancement of post-glomerular vasodilatation in patients with T2D, as illustrated in Fig. 8b.

## Are the effects of SGLT2 inhibitors on renal hemodynamics in T1D and T2D different?

While the effects in the T1D in young adults revealed reduction of hyperfiltration and intraglomerular pressure by vasoconstriction of afferent arterioles, the T2D study in older adults revealed efferent arteriolar vasodilation as the mediator of the decrease in GFR and intraglomerular pressure. These are remarkably different effects of SGLT2 inhibitors in two different patient groups with diabetes. What could be the reason for these differences? First of all, there are important differences between the patient cohorts of the two studies that are beyond the difference of the T1D and T2D pathogenesis. These include age, glycemic control, blood pressure, and renal function. All these parameters could affect difference in SGLT2 inhibitor effects on renal hemodynamics. Notably, the number of nephrons declines with age (by about 50% in healthy individuals between 20 and 70 years of age) while the single-nephron GFR in the remaining glomeruli varies only slightly [32]. Therefore, the differences observed in the two studies may be in part a reflection of variability in nephron numbers among the patient cohorts. Also, there are great renal structural heterogeneities due to diabetic nephropathy in older T2D patients compared with younger T1D patients. In addition, the T2D study comprised a combination therapy with an ARB drug.

### Clinical benefits in T1D and T2D patients

Taken together, SGLT2 inhibitors protect against major adverse kidney outcomes in individuals with T1D and T2D. In addition, they prevent kidney failure and reduce morbidity in patients with T2D. Combined therapies in T2D with other drugs such as ARBs prove effective for added nephroprotective actions. Combination therapies with SGLT2 inhibitors were found to be relatively free of complications, as shown by meta-analyses of data from large clinical studies [112]. In addition, SGLT2 inhibitors turn out to have general nephroprotective effects, independent of diabetes. Overall, beneficial effects include lowering arterial blood pressure, improving blood sugar levels, reduction in blood uric acid levels, and slowing down progression of diabetic nephropathy.

### Renal-cardiac benefits of SGLT2 inhibitors

SGLT2 inhibitors exhibit great benefits also for patients with heart failure but the precise mechanism underlying this renal-cardiac benefit are not completely understood. Renal autoregulation could contribute toward the observed favorable effects in patients with heart failure with reduced ejection fraction (HFrEF), with and without diabetes, a topic that is currently under evaluation in clinical trials [91]. In addition, a variety of accompanied favorable changes may contribute toward these benefits: improving the blood glucose level, lowering arterial blood pressure, decrease in body weight and arterial blood volume, and reduction in uric acid levels. However, none of these factors fully explains the renal-cardiac benefits. Recently, direct effects of SGLT2 inhibitors on cardiomyocytes have been discussed although it is unclear what physiological role Na^+^-coupled glucose transporters play in these cells. Indeed, a recent report reveals that SGLT1 is expressed in cardiomyocytes from human tissue biopsies at both the RNA and protein levels. Interestingly, the study revealed that, while there was no expression of SGLT2, SGLT1 was expressed in normal myocardial tissue and significantly upregulated in ischemia and hypertrophy [33]. The increase in SGLT1 expression in ischemic and hypertrophic myocardium was associated with increased phosphorylation in activating domains of the intracellular second messengers ERK1/2 and mTOR, thereby mediating PKC-stimulated plasma membrane expression of SGLT1, representing a potential pharmacological target for cardio-protection. However, SGLT2 inhibitors are poor ligands for SGLT1. Thus, off-target effects of SGLT2 inhibitors that are not related to membrane transporters in cardiomyocytes cannot be completely excluded in this study.

### Safety of SGLT2 inhibitors and adverse effects

Although there is concern that genital infections are common adverse effects of these inhibitors, with mycotic infections, urinary tract infections, and osmotic diuresis, meta-analyses of trials and a large population-based cohort study indicated that there is no increased risk, providing reassurance for patients [199]. Since SGLT2 inhibitors are, however, a fairly new class of drugs, information on long-term adverse effects are not available yet. The safety of SGLT2 inhibitors is, however, warranted due to their highly SGLT2-selective nature. As indicated above, SGLT2-selective inhibitors do not significantly inhibit intestinal SGLT1, which is important to prevent diarrhea that would be caused when SGLT1 would be highly inhibited, similar to glucose/galactose malabsorption [178]. In the kidney, SGLT2-selectivity is also important from the safety point of view. When SGLT2 in the proximal portion of the renal proximal tubules is completely inhibited, downstream SGLT1 in the distal portion of the proximal tubule compensates for the glucose reabsorption. When SGLT1 is fully functional, it can reabsorb 120 g of glucose per day, whereas under euglycemic conditions, it is responsible for the reabsorption of only 20 g of glucose per day [1]. This compensation of glucose reabsorption by SGLT1 is important to prevent hypoglycemia when using SGLT2 inhibitors for the treatment of diabetic patients. In fact, the individuals with familial renal glucosuria, a genetic loss of SGLT2 function, do not generally suffer from hypoglycemia, an observation which supports the safety of the use of SGLT2 inhibitors [186]. Therefore, it is crucial that SGLT2 inhibitors do not inhibit SGLT1 in the renal proximal tubules. Based on the plasma concentration and protein binding of the SGLT2 inhibitor canagliflozin, it was in fact estimated that it does not affect SGLT1 in proximal tubules where the drug inhibits SGLTs by binding them from the luminal side [107, 116]. Similarly, SGLT2 inhibitors are expected not to affect SGLT1 expressed in the heart and skeletal muscle when used at clinical doses, which would further support the safety of the drugs [116].

The highly SGLT2-selective nature has been an important requirement for SGLT2 inhibitors as described above. Therefore, SGLT2 inhibitors with high selectivity that are not affecting other SGLTs have been developed and launched as therapeutic drugs [6]. However, it has been recognized that α-glucosidase inhibitors that eventually suppress glucose absorption from the small intestine are effective in the control of postprandial hyperglycemia that most anti-diabetic agents are not able to normalize, even though they reduce fasting blood glucose levels [8]. Because the inhibition of glucose absorption from the small intestine would have a beneficial contribution to the anti-diabetic action, the partial inhibition of SGLT1 in the small intestine without causing diarrhea has been considered. Among SGLT2 inhibitors currently used clinically, canagliflozin shows less selectivity to SGLT2 [89]. Furthermore, because canagliflozin shows the highest plasma protein binding, a higher dose is set for clinical usage [116]. Therefore, canagliflozin, when administered orally, inhibits glucose absorption from the upper small intestine transiently and partially, which does not cause diarrhea but contributes to reduce postprandial hyperglycemia [107, 116, 128]. Furthermore, the transient, partial inhibition of glucose absorption from the upper small intestine causes a partial shift of glucose absorption from the upper to lower small intestine and eventually increases glucose absorption from the lower small intestine, which increases the secretion of incretin GLP-1 from the lower small intestine [128]. This also contributes to anti-diabetic action of canagliflozin, which is the characteristic feature of canagliflozin among SGLT2 inhibitors.

The effect of SGLT2 inhibitors on bone fractures has recently been discussed. Evidence indicating the direct effect of SGLT2 inhibitors on fracture risk is lacking and an increased number of falls probably contributes to fractures [131]. SGLT2 inhibitors might indirectly increase bone turnover by weight loss. Determining the relevance of the effect of SGLT2 inhibitors on bone fractures and mineral metabolism in T2D, however, requires further investigation [189].

## Future perspectives

While the transport mechanisms and physiological roles of human SGLT1 in intestine and kidney have been extensively studied, there are emerging observations for new roles in other tissues that require further attention. These include expression in the heart, where its role is completely unknown or in the brain where it is proposed contribute to glucose sensing. Some of the SGLT2 inhibitors can also interact with SGLT1 and, thus, while being beneficial for diabetes treatment, they could affect some of these other functions as well. Also, while SGLT2 expression was thought to be largely confined to the early proximal tubules of the kidney, recent studies reveal expression in certain cancer types as well. This opens new opportunities for the development of cancer therapies using SGLT2 inhibitors, thereby blocking delivery of glucose into cancer cells and impairing energy production.

More studies are also needed to uncover the physiological roles of other members of the SLC5 family. Suitable experimental tools are needed to define the true role of SGLT3, a potentially interesting protein that might act as a glucose sensor. However, further examination of this hypothesis is still needed. Also, only sparse information is available on SGLT4, a transporter that is particularly interesting as it seems to reabsorb the metabolite 1,5-anhydroglucitol in the kidney. Since an FDA-approved clinical test for diabetes patients is in use that might depend on the functional activity of SGLT4, further studies of the role of this transporter in health and disease would be valuable. Also, information on SGLT5 is still very limited. The expression pattern of both SGLT4 and SGLT5 in intestine and kidney somewhat resembles that of SGLT1 and SGLT2. However, they do not appear to function as backup Na^+^-dependent glucose transporters and they exhibit different sugar specificities compared with SGLT1 and SGLT2. Therefore, it will be important to clarify their specific contributions toward solute transport in intestine and kidney.

While the existing bacterial vSGLT and SiaT structures have contributed significantly to our understanding of the mechanism of inhibition of gliflozins, their detailed action on human SGLTs is still unknown. Structural biology efforts to elucidate the atomic structures of human SGLTs in complex with substrates and/or inhibitors are warranted. Such structures could also help to answer the question to what extent the selectivity of these compounds depends on particular dynamic properties of SGLTs or the interaction of the inhibitors with specific protein side-chains.

Identifying further the precise mechanisms of the effects of SGLT2 inhibition on renal hemodynamics and how they differ in T1D and T2D patients, as well as further uncovering the regulation mechanisms of SGLT2 upregulation in early renal proximal tubules in the diabetic states and the compensatory mechanisms of upregulation of SGLT1 in late renal proximal tubules in the absence of SGLT2, may provide a foundation for future drug discovery and strategies for novel patient-tailored therapies, including combination therapies.
